# Deep Electronic State Regulation through Unidirectional Cascade Electron Transfer Induced by Dual Junction Boosting Electrocatalysis Performance

**DOI:** 10.1002/advs.202304063

**Published:** 2023-09-15

**Authors:** Wenlin Zhang, Chonghong Shu, Jiayu Zhan, Shenghu Zhang, Lu‐Hua Zhang, Fengshou Yu

**Affiliations:** ^1^ National‐Local Joint Engineering Laboratory for Energy Conservation in Chemical Process Integration and Resources Utilization School of Chemical Engineering and Technology Hebei University of Technology Tianjin 300130 P. R. China

**Keywords:** dual junction, electrochemical O_2_ reduction, electronic state regulation, interface engineering, unidirectional cascade electron transfer

## Abstract

Unidirectional cascade electron transfer induced by multi‐junctions is essential for deep electronic state regulation of the catalytic active sites, while this advanced concept has rarely been investigated in the field of electrocatalysis. In the present work, a dual junction heterostructure (FePc/L‐R/CN) is designed by anchoring iron phthalocyanine (FePc)/MXene (L‐Ti_3_C_2_‐R, R═OH or F) heterojunction on g‐C_3_N_4_ nanosheet substrates for electrocatalysis. The unidirectional cascade electron transfer (g‐C_3_N_4_ → L‐Ti_3_C_2_‐R → FePc) induced by the dual junction of FePc/L‐Ti_3_C_2_‐R and L‐Ti_3_C_2_‐R/g‐C_3_N_4_ makes the Fe center electron‐rich and therefore facilitates the adsorption of O_2_ in the oxygen reduction reaction (ORR). Moreover, the electron transfer between FePc and MXene is facilitated by the axial Fe─O coordination interaction of Fe with the OH in alkalized MXene nanosheets (L‐Ti_3_C_2_‐OH). As a result, FePc/L‐OH/CN exhibits an impressive ORR activity with a half‐wave potential (*E*_1/2_) of 0.92 V, which is superior over the catalysts with a single junction and the state‐of‐the‐art Pt/C (*E*_1/2_ = 0.85 V). This work provides a broad idea for deep regulation of electronic state by the unidirectional cascade multi‐step charge transfer and can be extended to other proton‐coupled electron transfer processes.

## Graphical abstract

The unidirectional cascade charge transfer induced by the dual junction is demonstrated to significantly boost the electrochemical oxygen reduction reaction (ORR) performance. The directional transfer of electrons through interfaces can deeply regulate the electronic state of the Fe site, enabling FePc/L‐OH/CN to have superior ORR performance over the catalysts with the single junction and the state‐of‐the‐art Pt/C.

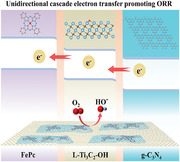

## 1 Introduction

Oxygen reduction reaction (ORR) involves multi‐step electron transfer and produces slow kinetics that hinders the commercial application of energy storage and conversion apparatus, including metal‐air batteries and fuel cells.^[^ ^1^, ^2^, ^3^, ^4^, ^5^, ^6^ ^]^ The development of advanced electrocatalysts capable of fast electron transfer is a daunting task, and in recent years, the chemically engineered hetero‐structured electrocatalysts with modulated interfaces have proven to be effective for boosting the electrocatalytic performance of ORR.^[^ ^7^, ^8^, ^9^, ^10^ ^]^ It is well known that the formation of Mott‐Schottky heterojunctions and semiconductor junctions (*n*‐*n*, *p*‐*n*, and *p*‐*p* junctions) can induce electronic reconfiguration at the heterogeneous interface, modulating the electronic state of the active site and accelerating the charge transfer rate, thus significantly increasing the inherent activity of catalysts.^[^ ^11^, ^12^, ^13^, ^14^, ^15^ ^]^ For instance, Ding et al. developed a novel jellyfish‐shaped catalyst, and the intrinsic catalytic activity of this catalyst was boosted by electron redistribution at the heterojunction interface.^[^ ^16^ ^]^ Qian et al. demonstrated that the spontaneous charge redistribution at the FeNi‐LDH/CoP heterogeneous interface is essential to promote charge transfer capability and lower the oxygen electrocatalytic reaction potential, which ultimately improves the intrinsic activity of catalysts.^[^ ^17^ ^]^ While the single junction provides a limited regulation degree of charge density,^[^ ^18^ ^]^ and therefore the deep interfacial electron redistribution is desired for further facilitation of catalytic performance.

The construction of multi‐junction enables the relatively large driven force for charge transfer and provides a unidirectional cascade electron transfer channel, which can greatly accelerate the separation and transfer of interfacial charges. The rational design of multi‐component catalyst systems with cascade electron transfer is highly desirable for electron state regulation and has been widely used in photocatalysis.^[^ ^19^, ^20^, ^21^, ^22^, ^23^, ^24^ ^]^ For instance, Jing et al. constructed a novel ternary heterojunction photocatalyst (T‑CN/BVNS), leading to a high selectivity for CO_2_‐to‐CO conversion through forming an efficient cascaded charge transfer channel.^[^ ^25^ ^]^ While the design of ternary catalysts with unidirectional cascaded electron transfer channels remains a significant challenge because the third component may be randomly spatially distributed on the surface of the other two components leading to the formation of multiple independent electron transfer pathways. The 2D‐2D heterogeneous structure has a larger interfacial contact area and stronger interaction between the two components, and the face‑to‑face strong heterogeneous interfacial coupling makes it easier to construct unidirectional cascade electron transfer channels and enhance interfacial charge transfer. This is conducive to the rearrangement of the electronic structure of the active site, thus contributing to the improvement of catalytic performance.^[^ ^26^ ^]^ However, to the best of our knowledge, this advanced concept has rarely been explored in the field of electrocatalysis.

Herein, we designed the ternary heterostructure (FePc/L‐R/CN) catalysts with unidirectional cascade electron transfer (g‐C_3_N_4_ → L‐Ti_3_C_2_‐R → FePc) by building FePc/L‐Ti_3_C_2_‐R (R═OH or F) heterojunction on g‐C_3_N_4_ nanosheet substrates. The unidirectional electron transfer induced by the dual junction between FePc, MXene nanosheets, and g‐C_3_N_4_ nanosheets accelerates the charge separation and directional transfer, making the active site Fe center electron‐rich and promoting the adsorption of O_2_, which is the key to facilitate electrocatalytic oxygen reduction. Moreover, the electron transfer of FePc and MXene was facilitated by the axial Fe‐O coordination interaction between Fe with the OH in alkalized MXene nanosheets (L‐Ti_3_C_2_‐OH). As a result, FePc/L‐OH/CN exhibits an impressive ORR activity with an onset potential (*E*_0_) of 1.02 V and a half‐wave potential (*E*_1/2_) of 0.92 V, which is superior over the catalysts with single junction and the state‐of‐the‐art Pt/C (*E*_0_ = 1.01 V, *E*_1/2_ = 0.85 V). The design provides a valuable research reference for deep regulation of electronic state by the unidirectional cascade multi‐step charge transfer and can be extended to other proton‐coupled electron transfer processes.

## 2 Results and Discussion

**Figure 1:**
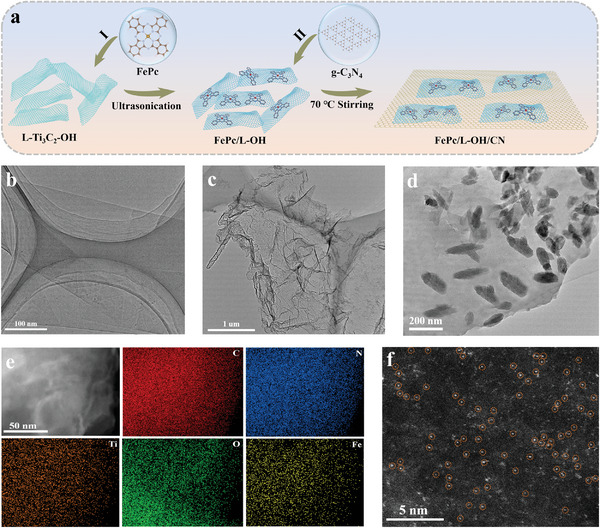
a) Schematic diagram for the synthesis of the FePc/L‐OH/CN catalyst. TEM images of b) L‐Ti_3_C_2_‐OH, c) g‐C_3_N_4_, and d) FePc/L‐OH/CN. e) HAADF‐STEM image of FePc/L‐OH/CN with mappings of each element. f) AC‐HAADF‐STEM image of FePc/L‐OH/CN.

A simple self‐assembly strategy was implemented to synthesize FePc/L‐R/CN catalysts containing the dual junction (**Figure** **1**a). Initially, a suspension of surface‐functionalized MXene nanosheets (L‐Ti_3_C_2_‐R, R═OH or F) was prepared by etching, stripping, and functionalization processes (Figures S1 and S2, Supporting Information). Then a certain amount of FePc was covalently grafted onto L‐Ti_3_C_2_‐R. Finally, the as‐prepared composite was uniformly dispersed on the g‐C_3_N_4_ nanosheets by electrostatic interactions, forming the FePc/L‐R/CN catalysts.

Transmission electron microscopy (TEM) images show that the as‐prepared L‐Ti_3_C_2_‐OH material exhibits a typical nanosheet morphology (Figure 1b). In the high‐resolution TEM (HR‐TEM) image of L‐Ti_3_C_2_‐OH (Figure S3a, Supporting Information), clear streaks with a planar spacing of 0.216 nm were found, corresponding to the (105) plane of the monolayer structure.^[^ ^27^ ^]^ An average sample thickness of ≈1.88 nm (Figure S3b, Supporting Information) is consistent with the 1–2 nm thickness of monolayer Ti_3_C_2_T_x_ nanosheets.^[^ ^28^ ^]^ X‐ray photoelectron spectroscopy (XPS) (Figure S4, Supporting Information) and Raman spectroscopy (Figure S5a, Supporting Information) documented the changes of the terminating groups on the surface of L‐Ti_3_C_2_T_x_ after surface functionalization. Fourier transform infrared spectroscopy (FT‐IR) further verified the substitution of the surface‐terminating groups (Figure S5b, Supporting Information), and relative to unfunctionalized L‐Ti_3_C_2_T_x_, no obvious C‐F signal was detected for L‐Ti_3_C_2_‐OH and the ─OH signal was significantly weakened for L‐Ti_3_C_2_‐F, which are consistent with the XPS and Raman analysis.

The ternary heterostructure (FePc/L‐R/CN) was constructed by building FePc/L‐Ti_3_C_2_‐R (R═OH or F) heterojunction nanosheets on g‐C_3_N_4_ nanosheets substrate (Figure 1c). FePc/L‐R/CN shows a classic nanosheet structure with the uniform dispersion of L‐Ti_3_C_2_‐OH on g‐C_3_N_4_ nanosheets (Figure 1d). The elements of Fe, Ti, C, N, and O in FePc/L‐OH/CN were homogeneously dispersed throughout the sample revealed by energy‐dispersive X‐ray spectroscopy (EDS) mapping (Figure 1e). To reveal more clearly the dispersion of iron species, the aberration‐corrected high‐angle annular dark‐field scanning transmission electron microscopy (HAADF‐STEM) was also carried out, which shows the uniform individual bright spots assigned to the isolated Fe atoms (Figure 1f). The Fe content in FePc/L‐OH/CN analyzed by inductively coupled plasma mass spectrometry (ICP‐MS) was 0.90%. We can observe the characteristic diffraction peaks of FePc, L‐Ti_3_C_2_‐R, and g‐C_3_N_4_ in the X‐ray diffraction (XRD) pattern of FePc/L‐R/CN (Figure S6, Supporting Information), confirming the formation of ternary heterostructures.^[^ ^29^ ^]^ The similar FT‐IR spectra of FePc/L‐R/CN and g‐C_3_N_4_ indicated that the integration of FePc and L‐Ti_3_C_2_‐R shows negligible effect in structure for the internal g‐C_3_N_4_ and the uniform dispersion of FePc/L‐R on g‐C_3_N_4_ nanosheets consistent with the XRD analysis (Figure S7, Supporting Information).^[^ ^30^ ^]^ Compared with FePc/L‐OH, FePc/L‐OH/CN shows a typical IV‐type isothermal curve with a significant hysteresis return line and an increased number of mesopores (Figure S8, Supporting Information), which provided a larger specific surface area and higher porosity to promote electrolyte transport.^[^ ^31^ ^]^ The anchor of FePc on L‑Ti_3_C_2_‐OH rather than g‐C_3_N_4_ nanosheet was confirmed by the similar N 1s spectra and the obvious shift in the binding energy of Ti 2p spectra for FePc/L‐OH/CN and L‑OH/CN (Figures S9 and S10, Supporting Information).^[^ ^32^ ^]^

**Figure 2:**
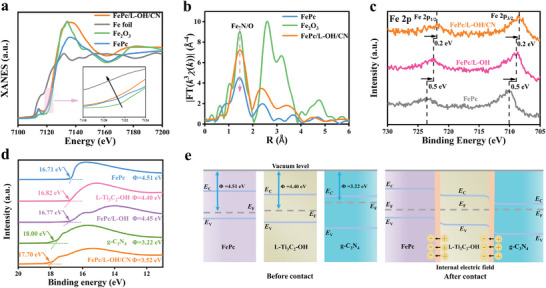
a) XANES spectra at Fe K‐edge of FePc/L‐OH/CN, FePc, Fe, and Fe_2_O_3_. b) EXAFS spectra at Fe K‐edge of FePc/L‐OH/CN, FePc, and Fe_2_O_3_. c) XPS spectra of Fe 2p for FePc, FePc/L‐OH, and FePc/L‐OH/CN. d) UPS spectra of FePc, L‐Ti_3_C_2_‐OH, FePc/L‐OH, g‐C_3_N_4_, and FePc/L‐OH/CN. e) Schematic illustration of the FePc, L‐Ti_3_C_2_‐OH, and g‐C_3_N_4_ before and after contact.

Compared with FePc/L‐F, a larger proportion of Fe─O bond can be observed in FePc/L‐OH, implying the axial coordination interaction of FePc to L‐Ti_3_C_2_‐OH (Figure S11, Supporting Information). X‐ray absorption near edge structure (XANES) and extended X‐ray absorption fine structure (EXAFS) measurements were performed to analyze the coordination environment and electronic state of the Fe sites. In the XANES of Fe K‐edge (**Figure** **2**a), a significant negative shift in FePc/L‑OH/CN relative to FePc was observed, indicating a change in the electronic state of Fe sites. In addition, a shoulder peak at 7115 eV was assigned to the D_4h_ symmetric square planar configuration of FePc. In contrast, the intensity of this peak was reduced for FePc/L‐OH/CN, which was possibly induced by the additional coordination of OH to Fe breaking the symmetric structure of the FeN_4_ plane.^[^ ^33^ ^]^ Additionally, the EXAFS spectra of the Fe K‐edge show that the number of Fe coordination in FePc/L‐OH/CN is higher than the exact tetra‐coordination in FePc (FeN_4_) and lower than the exact hexa‐coordination in Fe_2_O_3_ (FeO_6_) (Figure 2b), which implies that there may be one additional coordination between FePc and OH in FePc/L‐OH/CN.^[^ ^34^ ^]^ The wavelet transform (WT) data show that FePc/L‐OH/CN exhibits the maximum WT intensity at 3.46 Å^−1^ (Figure S12, Supporting Information), which is clearly different from FePc (3.26 Å^−1^) and closer to Fe_2_O_3_ (3.58 Å^−1^), further indicating the possible existence of Fe─O coordination. By fitting the EXAFS curves in R‐space and K‐space (Figure S13 and Table S1, Supporting Information), the experimental data were found to be highly consistent with the conformation of FeN_4_O_1_. These results demonstrate that in FePc/L‐OH/CN, an additional axial Fe─O coordination is formed between FePc and L‐Ti_3_C_2_‐OH.

With the confirmation for the assembly of FePc/L‐R/CN, the dual junction and electron transfer pathway were investigated. Both C 1s and N 1s XPS spectra of FePc/L‐R/CN show an obvious shift toward higher binding energy compared to g‑C_3_N_4_, demonstrating that the heterojunction was constructed between FePc/L‐R and g‐C_3_N_4_ (Figure S14, Supporting Information). For the Fe 2p XPS spectra, the binding energy of FePc/L‐OH shows a negative shift of 0.5 eV compared with the pristine FePc (Figure 2c), indicating a heterojunction with strong interactions between FePc and L‐Ti_3_C_2_‐OH was built. This shift degree is larger than that for FePc/L‐F (0.2 eV, Figure S15, Supporting Information) because the axial Fe─O coordination ensures strong electron interactions for enhanced high‐speed electron transport. Moreover, the heterojunction between FePc/L‐R and g‐C_3_N_4_ accelerates the separation and transfer of interfacial charges, further increasing the charge density around the Fe site (Figure 2c; Figure S15, Supporting Information). To further determine the electron transfer pathways between g‐C_3_N_4_, L‐Ti_3_C_2_‐OH, and FePc interfaces, ultraviolet photoelectron spectroscopy (UPS) was performed. The work functions were calculated to be 3.22, 4.40, 4.51, 4.45, and 3.52 eV for g‐C_3_N_4_, L‐Ti_3_C_2_‐OH, FePc, FePc/L‐OH, and FePc/L‐OH/CN (Figure 2d), respectively.^[^ ^35^ ^]^ This result similarly demonstrated the existence of heterojunctions between FePc and L‐Ti_3_C_2_‐OH, and between L‐Ti_3_C_2_‐OH and g‐C_3_N_4_ forming a unidirectional cascade electron transfer channel (Figure 2e), where the charge can be spontaneously transferred (g‐C_3_N_4_ → L‐Ti_3_C_2_‐OH → FePc), making the Fe site electron‐rich and further affecting the electronic state of Fe.^[^ ^36^ ^]^

**Figure 3:**
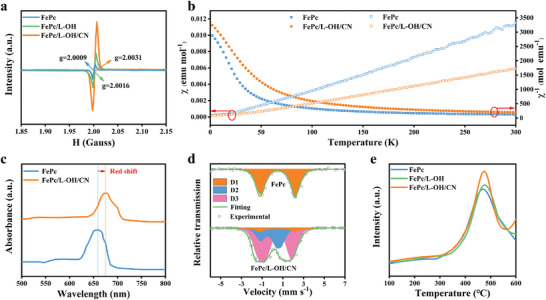
a) The EPR spectra with *g*‐factor value of FePc, FePc/L‐OH, and FePc/L‐OH/CN. b) *χ*_m_ and 1/χ_m_ plots of FePc and FePc/L‐OH/CN. c) The UV‐vis spectra of FePc and FePc/L‐OH/CN. d) ^57^Fe Mussbauer transmission spectra of FePc and FePc/L‐OH/CN. e) The oxygen adsorption‐desorption test.

The electronic state of the Fe center in FePc/L‐OH/CN was further analyzed using electron paramagnetic resonance (EPR) spectroscopy, temperature‐dependent magnetization (*M*‐*T*) measurements, and UV‐vis spectroscopy. In the EPR spectra, FePc/L‐OH/CN shows an obvious increase in the *g* value compared to FePc, L‐Ti_3_C_2_‐OH, and FePc/L‐OH (**Figure** **3**a; Figure S16, Supporting Information), indicating an increase in the number of unpaired electrons around the Fe site and the possible change in the electronic state.^[^ ^37^ ^]^ *M*‐*T* analysis shows that the introduction of the unidirectional cascade electronic channel enhances the paramagnetic state of the Fe site (Figure 3b). This provides strong evidence for more free electrons with bubbly paramagnetic properties moving around the Fe atom.^[^ ^38^ ^]^ UV‐vis analysis shows a red shift in the Q‐band of FePc/L‐OH/CN compared to FePc (Figure 3c), indicating a decrease in the electron leap energy, which in turn may affect the electronic state of the Fe center.^[^ ^39^ ^]^ The effect of electronic regulation for Fe on O_2_ adsorption behavior was investigated by ^57^Fe Mussbauer spectra and O_2_ temperature‐programmed desorption (O_2_‐TPD) test. For ^57^Fe Mussbauer spectra, a smaller D1 bimodal peak and two distinct D2 and D3 bimodal peaks were observed for FePc/L‐OH/CN (Figure 3d). The D1, D2, and D3 bimodal peaks were attributed to planar symmetric FeN_4_ species, O‐FeN_4_ species, and O‐FeN_4_ with surface‐adsorbed O_2_ molecules site (O‐FeN_4_‐O_2_). In contrast, there is only one D1 bimodal peak for FePc, with no obvious O_2_ adsorption signal.^[^ ^40^ ^]^ The enhanced O_2_ adsorption of FePc/L‐OH/CN was further confirmed by a stronger O_2_ desorption peak intensity in the O_2_‐TPD test (Figure 3e). These results consistently confirm that the relatively high electron density in the Fe site induced by the directional transfer of interfacial charges (g‐C_3_N_4_ → L‐Ti_3_C_2_‐OH → FePc) can promote O_2_ adsorption, the prelude for ORR.

**Figure 4:**
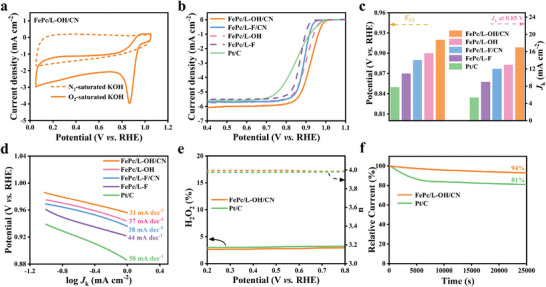
a) CV curves of FePc/L‐OH/CN. b) LSV curves of FePc/L‐OH/CN, FePc/L‐F/CN, FePc/L‐OH, FePc/L‐F, and Pt/C. c) Values of *E*_1/2_ and *J*_K_ at 0.85 V of FePc/L‐OH/CN, FePc/L‑F/CN, FePc/L‐OH, FePc/L‐F, and Pt/C. d) Tafel slope of FePc/L‐OH/CN, FePc/L‐F/CN, FePc/L‐OH, FePc/L‐F, and Pt/C. e) H_2_O_2_ yield and number of electrons transferred in FePc/L‑OH/CN and Pt/C. f) chronoamperometric test curves of FePc/L‐OH/CN and Pt/C.

The performance of electrocatalytic ORR with FePc/L‐OH/CN was initially evaluated by cyclic voltammetry (CV) measurements in a typical three‐electrode system. Compared with in N_2_ saturated KOH solution, FePc/L‐OH/CN shows an obvious redox peak in O_2_ saturated KOH solution (**Figure** **4**a), demonstrating an effective electrocatalytic ORR performance.^[^ ^41^, ^42^ ^]^ Then LSV tests were further performed to evaluate the ORR electrocatalytic performance (Figure 4b). The onset potential (*E*_0_) and half‐wave potential (*E*_1/2_) of FePc/L‐OH/CN were 1.02 and 0.92 V, respectively, which was better than Pt (*E*_0_ = 1.01 V, *E*_1/2_ = 0.85 V), FePc/L‐F/CN (*E*_0_ = 0.97 V, *E*_1/2_ = 0.89 V), FePc/L‐OH (*E*_0_ = 0.98 V, *E*_1/2_ = 0.90 V), and FePc/L‐F (*E*_0_ = 0.96 V, *E*_1/2_ = 0.87 V). The superior ORR performance of FePc/L‐OH/CN is comparable to most of the individual iron site catalysts reported in the recent literature (Table S2, Supporting Information). When FePc was directly loaded on g‐C_3_N_4_, FePc/g‐C_3_N_4_ maintains the nanosheet state of pristine g‐C_3_N_4_, and the electrons are transferred from g‑C_3_N_4_ to FePc (Figure S17a–c, Supporting Information). The half‐wave potential of FePc/g‐C_3_N_4_ is only 0.86 V (Figure S17d, Supporting Information). Moreover, when g‐C_3_N_4_ was replaced with a conductor, i.e., graphene (G) to construct the ternary heterostructure (FePc/L‐OH/G). No obvious electron transfer was detected in the G and L‐Ti_3_C_2_‐OH interface (Figure S18a,b, Supporting Information). Compared with Fe/L‐OH (*E*_0_ = 0.98 V, *E*_1/2_ = 0.90 V), FePc/L‐OH/G shows a very similar ORR performance (*E*_0_ = 0.98 V, *E*_1/2_ = 0.908 V) (Figure S18c, Supporting Information). The slight improvement in *E*_1/2_ for FePc/L‐OH/G may be induced by the uniform dispersion of FePc/L‐OH on G. These results further indicate that the unidirectional cascade electron transfer induced by the dual junction plays a crucial role in electrocatalytic ORR.

The enhanced catalytic performance was also reflected by the kinetic current density, FePc/L‐OH/CN catalyst shows a 17 mA cm^−2^ current density at 0.85 V, which was 3.1 times higher than that of Pt/C (5.4 mA cm^−2^) and higher than that of the other comparison samples, with a trend consistent with the potential (Figure 4c).^[^ ^43^, ^44^ ^]^ As expected, FePc/L‐OH/CN shows a favorable Tafel slope of 31 mV dec^−1^ (Figure 4d), indicating a fast ORR kinetic process.^[^ ^45^ ^]^ With the electrochemical impedance spectroscopy test (Figure S19, Supporting Information), FePc/L‐OH/CN displays the smallest charge transfer resistance, further indicating the dual junction can enhance the reaction kinetics. The double layer capacitance values (*C*_dl_) (Figure S21, Supporting Information) proportional to the electrochemically active surface area (ECSA) were calculated through CV testing (Figure S20, Supporting Information), the FePc/L‐OH/CN exhibits the largest electrochemically active surface area with large exposure of accessibly active sites.^[^ ^46^ ^]^ We normalized the *J*_K_ of all samples using ECSA extracted from the bilayer capacitance (*C*_dl_). The ECSA normalized activity of FePc/L‐OH/CN was higher than that of the other comparison samples (Figure S22, Supporting Information), indicating that FePc/L‐OH/CN has a higher intrinsic catalytic activity for ORR. To further analyze the ORR reaction mechanism of FePc/L‐OH/CN catalyst, the average number (*n*) of electrons transferred during the ORR of FePc/L‐OH/CN was determined to be about four based on the *K*‐*L* equation of LSV at different rotational numbers (Figure S23, Supporting Information). The H_2_O_2_ yield and number of electrons transferred were further investigated by rotating ring disc electrode (RRDE) experiments (Figure 4e). The number of electrons transferred of FePc/L‐OH/CN is close to four, which is consistent with the *K*‐*L* equation results, and the hydrogen peroxide selectivity is kept below 3%, indicating that the ORR on FePc/L‐OH/CN is a more efficiently four‐electron transfer pathway. An accelerated durability experiment confirmed that FePc/L‐OH/CN is an excellent ORR electrocatalyst (Figure S24, Supporting Information), and after 2000 redox cycles, the *E*_1/2_ was only negatively shifted by 9 mV, significantly <21 mV for Pt/C. After an amperometric I‐t test at 25 000 s (Figure 4f), the FePc/L‐OH/CN shows a relative current retention of 94%, which exceeded that of Pt/C by 81%. Additionally, the current decreased slightly after the addition of methanol (Figure S25, Supporting Information), while the current of Pt/C decreased substantially, revealing that FePc/L‐OH/CN shows an excellent methanol tolerance.

**Figure 5:**
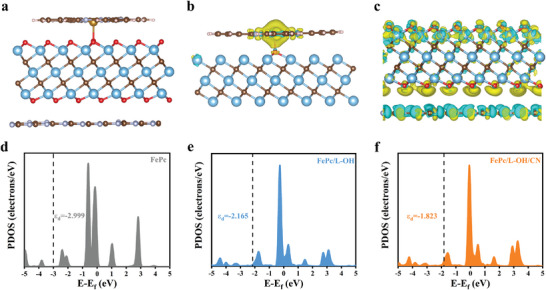
a) The theoretical model of FePc/L‐OH/CN. The differential charge density map of b) FePc/L‐OH and c) L‐OH/CN. The yellow and cyan iso‐surfaces represent electron density gain and loss, respectively. The partial density of states (PDOS) for Fe 3d of d) FePc, e) FePc/L‐OH, and f) FePc/L‐OH/CN.

To further reveal the electronic state regulation, density‐functional theory (DFT) calculations were carried out (**Figure** **5**a). The electron transfer behavior was revealed quantitatively by calculating the obtained differential charge densities (Figure 5b,c).^[^ ^47^ ^]^ The dual junction induced charge redistribution at the interface and triggered the unidirectional cascade electron transfer (g‐C_3_N_4_ → L‑Ti_3_C_2_‐OH → FePc), which is consistent with the XPS and UPS results. Then, we obtained the visualized charge distributions of the valence band maximum (VBM) and the conduction band minimum (CBM) by DFT calculations (Figure S26, Supporting Information). Obviously, after the introduction of g‐C_3_N_4_, the dual junction is formed, and orbital rearrangement occurs in VBM and CBM, which can produce a higher charge density, thus reducing the band gap and favoring electron transfer.^[^ ^48^ ^]^ We also calculated the partial density of states for FePc (−2.999 eV), FePc/L‐OH (−2.165 eV), and FePc/L‐OH/CN (−1.823 eV) (Figure 5d–f). Notably, when FePc is decorated with L‐Ti_3_C_2_‐OH, the single junction can regulate the electronic state leading to the d‐band center close to the Fermi energy level. More interestingly, when FePc/L‐OH is further loaded onto g‐C_3_N_4_, the d‐band center is brought closer to the Fermi energy level. These results support our conclusion that the dual junction can provide deep electronic state regulation, which results in superior electrocatalytic activity for ORR.

To explore the potential applications of FePc/L‐OH/CN, the practical performance was evaluated using a homemade zinc‐air battery (ZAB) (Figure S27a, Supporting Information). The ZAB with FePc/L‐OH/CN shows a stable open‐circuit voltage up to 1.5 V (Figure S27b, Supporting Information), closer to the theoretical value of 1.65 V than Pt/C. It also provides a higher peak power density (159 mW cm^−2^) (Figure S27c, Supporting Information), capacity (795 mA h g_Zn_^−1^) (Figure S27d, Supporting Information), and charge/discharge stability (150 h) (Figure S28, Supporting Information). As a demonstration, two zinc‐air batteries connected in series with FePc/L‐OH/CN as air cathodes could successfully light up red LEDs (Figure S29, Supporting Information), revealing their practical potential for power supply.

## 3 Conclusion

In summary, we have demonstrated that the unidirectional cascade charge transfer induced by the dual junction can significantly boost the electrocatalysis performance. The directional transfer through interfaces can deeply regulate the electronic state of the Fe site beneficial for O_2_ adsorption and activation. As a result, FePc/L‐OH/CN with dual junction exhibits superior performance over the catalysts with single junction and the state‐of‐the‐art Pt/C. This work provides a broad idea for deep regulation of electronic state by the unidirectional cascade multi‐step charge transfer and can be extended to other proton‐coupled electron transfer processes.

## Conflict of Interest

The authors declare no conflict of interest.

## Supporting information

Supporting Information

## Data Availability

The data that support the findings of this study are available from the corresponding author upon reasonable request.
