# Supplementary material for: Deep Electronic State Regulation through Unidirectional Cascade Electron Transfer Induced by Dual Junction Boosting Electrocatalysis Performance
Source: Adv Sci (Weinh). 2023 Sep 15;10(31):2304063. doi: 10.1002/advs.202304063 (PMC10625059; doi:10.1002/advs.202304063)
Supplement: Supplementary file 1 — Supporting Information [file ADVS-10-2304063-s001.pdf]

ADVANCED SCIENCE
Open Access

Supporting Information

for *Adv. Sci.*, DOI 10.1002/advs.202304063

Deep Electronic State Regulation through Unidirectional Cascade Electron Transfer Induced by Dual Junction Boosting Electrocatalysis Performance

Wenlin Zhang, Chonghong Shu, Jiayu Zhan, Shenghu Zhang, Lu-Hua Zhang and Fengshou Yu**

Supporting Information

Deep Electronic State Regulation through Unidirectional Cascade Electron Transfer Induced by Dual Junction Boosting Electrocatalysis Performance

Wenlin Zhang, Chonghong Shu, Jiayu Zhan, Shenghu Zhang, Lu-Hua Zhang, and Fengshou Yu**

Prof. W. Zhang, C. Shu, J. Zhan, S. Zhang, Prof. L.-H. Zhang, and Prof. F. Yu

National-Local Joint Engineering Laboratory for Energy Conservation in Chemical Process Integration and Resources Utilization, School of Chemical Engineering and Technology, Hebei University of Technology, Tianjin 300130, P. R. China.

E-mail: luhuazhang@hebut.edu.cn; yfsh@hebut.edu.cn

Methods

Chemicals.

Iron Phthalocyanine (FePc, 98%), dimethyl sulfoxide (DMSO, 99%), hydrofluoric acid (HF, 40%), melamine (98%), ethanol absolute (C_2H_5OH, AR), N, N-Dimethylformamide (DMF, AR) were purchased from Shanghai Aladdin Biochemical Technology Co., Ltd. Ti_3AlC_2 (MAX, 98%), sodium hydroxide (NaOH, 97%), potassium hydroxide (KOH, 98%), sodium Fluoride (NaF, 99%) were purchased by Shanghai Macklin Biochemical Co., Ltd. Commercial Pt/C (20 wt.%) was purchased from Suzhou Yilongsheng Energy Technology Co., Ltd. Ultrapure water was used throughout the experiment. All chemicals and solvents were used without further purification.

Synthesis of L-Ti_3C_2-R (R: -OH, -F) nanosheets.

Ti_3AlC_2 (1 g) was added to 20 mL of 40% hydrofluoric acid, and the mixture was stirred at 35 °C for 24 h to etch the metal aluminum completely. Next, the mixture was centrifuged at 3500 rpm and washed with deionized water until the supernatant pH=7, and the resulting precipitate was vacuum dried to obtain layered MXene (ML-$Ti_3C_2T_x$). ML-$Ti_3C_2T_x$ was dissolved in dimethyl sulfoxide (DMSO) and allowed to stand for 24 h to expand the interlayer spacing of ML-$Ti_3C_2T_x$. Then they were washed by centrifugation. ML-$Ti_3C_2T_x$ (100 mg) was added to 30 mL of deionized water and sonicated under N_2 protection for 1 h,

followed by centrifugation at 3500 r/min for 60 min to collect the dark green upper liquid containing the L-$Ti_3C_2T_x$ nanosheets. A certain amount of L-$Ti_3C_2T_x$ nanosheets solution was added to the NaOH solution, then stirred for 8 h under an N_2 atmosphere, centrifuged at 10 000 rpm, washed with water to pH=7, and finally freeze-dried to obtain L-Ti_3C_2-OH nanosheets. For comparison, L-Ti_3C_2-F was prepared by the same preparation method. The difference is that the NaOH solution was replaced with NaF.

Synthesis of g-C_3N_4 nanosheets.

A certain amount of melamine was put into a crucible with a lid and calcined at 550 °C for 4 h in a tube furnace with a heating rate of 2 °C/min. The yellow solid was obtained after natural cooling to room temperature. They were ground into powder and sieved through a standard 100-mesh sieve. Then, the solid was calcined at 550 °C for 2 h in a muffle stove with a heating rate of 5 °C/min giving g-C_3N_4 nanosheets.

Synthesis of FePc/L-R.

FePc (20 mg) and L-Ti_3C_2-R (20 mg) were dispersed in the 10 mL DMF solution under ultrasonic conditions, respectively. Next, FePc solution was poured into L-Ti_3C_2-R solution and the mixture was sonicated for 30 min. It was further stirred for 24 h to deepen the combination of FePc and L-Ti_3C_2-R. The solid was collected by suction filtration and washed with DMF and absolute ethanol. Then, the FePc/L-R was obtained by freeze-drying.

Synthesis of FePc/L-R/CN.

g-C_3N_4 (100 mg) was dispersed to 30 mL ethanol under ultrasonic conditions at room temperature, and FePc/L-R was added to the above solution under vigorous stirring. The mixture was then heated to 70 ºC and continuously stirred for 24 h. The solid was collected by filtration and washed several times with ethanol. Finally, the green FePc/L-R/CN was obtained by freeze-drying. The prepared sample was finally annealed at 120 °C for 1 h in an N_2 atmosphere to strengthen the interaction between substances.

Characterization.

Brunauer-Emmett-Teller (BET) tests were carried out using an ASAP-2460 gas adsorption analyzer at 77 K (Micromeritics Instrument Co., USA). Inductively coupled plasma mass spectrometry (ICP-MS) analysis was accomplished using Thermo ICAP Qc equipment (Thermo Fisher Scientific Co., USA) to obtain the loading mass of FePc. X-ray diffraction (XRD) was carried out to characterize the composition and crystal structures of the prepared materials. The Raman spectra were collected from a LabRam HR Evolution (Horiba) Raman spectrometer using a 532 nm laser. Fourier transform infrared (FT-IR) transmittance spectra were recorded using a Nicolet iS20 (Thermo Fisher Scientific Co., USA) IR spectrometer. X-ray photoelectron spectroscopy (XPS) was recorded on an ESCALAB 250 Xi spectrometer (Thermo Fisher Scientific Co., USA) with an Al Kα X-ray radiation. The Mössbauer spectra (^{57}Fe) were achieved by using an MS-500 instrument (Germany, Wissel) in transmission geometry with constant acceleration mode at room temperature. X-band Electron paramagnetic resonance (EPR) spectra were obtained by using a Bruker EMXplus spectrometer. Temperature magnetic susceptibility (M-T) curves were obtained by using a Quantum Design Squid-VSM physical property measurement system in the temperature region of 7 to 300 K to obtain the χ_m.

Electrochemical Measurements.

The electrochemical performance testing was carried out on the CHI 760E electrochemical analyzer using the classic three-electrode system. In the alkaline electrolyte, the glass carbon electrode (GCE) was used as the working electrode, and the Hg/HgCl electrode and the Pt electrode were considered as the reference electrode and the counter electrode, respectively. All of the measured potentials were converted into the reversible hydrogen electrode (RHE) scale. The sample (4 mg) was homogeneously dispersed in 1 mL of the mixed liquor of Nafion (5 wt.%) and ethanol, and then the ink was formed after 1 h of ultrasonic treatment. 10 μL ink was dropped on the surface of the polished glassy carbon electrode and dried at 25 °C. In the ORR experiment, the O_2 saturation was ensured by leading into a constant O_2 in the KOH solution.

Electrochemical data processing: Rotating ring-disk electrode (RRDE) measurements were performed at a scan rate of 5 mV s^{-1} at 1600 rpm, and the ring electrode voltage was kept at 1.3 V (*vs.* RHE).

The number of electrons transferred (n) and kinetic current density (J_K) during ORR were calculated according to Koutecky-Levich Equation:

$$\frac{1}{J}=\frac{1}{J_L}+\frac{1}{J_K}=\frac{1}{B\omega^{1/2}}+\frac{1}{J_K}$$

$$B=0.62nFC_0D_0^{2/3}\upsilon^{-1/6}$$

where J is the measured current, J_L is the diffusion-limited current densities, J_K is the kinetic-limited current densities, ω is the electrode rotation rate, n is the electron transfer number, B is the reciprocal of the slope, F is the Faraday constant (96485 C mol^{-1}), C_0 is the concentration of dissolved oxygen in the electrolyte (1.2×10^{-6} mol cm^{-3}), D_0 is the diffusion coefficient of dissolved oxygen (1.9×10^{-5} cm^2 s^{-1}), υ is the kinematic viscosity of the electrolyte (0.01 cm^2 s^{-1}).

The Hydrogen peroxide yield (H_2O_2%) and the electron transfer number (n) were determined by the following equations:

$$n=4\times\frac{I_D}{\frac{I_R}{N}+I_D}$$

$$H_2O_2(\%)=200\times\frac{I_D}{\frac{I_R}{N}+I_D}$$

where I_D is disk current, I_R is ring current, and N is the current collection efficiency of the Pt ring, which was provided as 0.37 by the manufacturer.

Zn-Air battery tests.

The gas diffusion electrode with a catalyst loading amount of 2.0 mg cm^{-2} is employed as a cathode in a homemade Zn-air batteries model. The aqueous solution of 6.0 M KOH (98%) and 0.2 M $Zn(OAc)_2$ was used as an electrolyte. The measurement of batteries was performed on the LAND testing system.

Computational details.

All calculations were carried out with the CP2K package (version 7.1) in the framework of the density functional theory (DFT), based on the hybrid Gaussian and plan-wave schemes.[1,2] Molecular orbitals of the valence electrons were expanded into DZVP-MOLOPT-SR-GTH basis sets, while atomic core

electrons are described through Goedecker-Teter-Hutter (GTH) pseudopotentials.[3,4] A plane-wave density cutoff of 800 Ry was adopted. The long-range van der Waals interaction is described by the DFT-D3 (BJ) approach.[5] All the structures were fully relaxed by CP2K with the BFGS scheme, and the force convergence criterion was set to 4.5 * 10^{-4} hartree/bhor. Due to the large numbers of atoms in the models, an orbital transformation (OT) scheme with only a gamma k-point was adopted during the calculations. The data processing was conducted using the VASPKIT code and VESTA package.

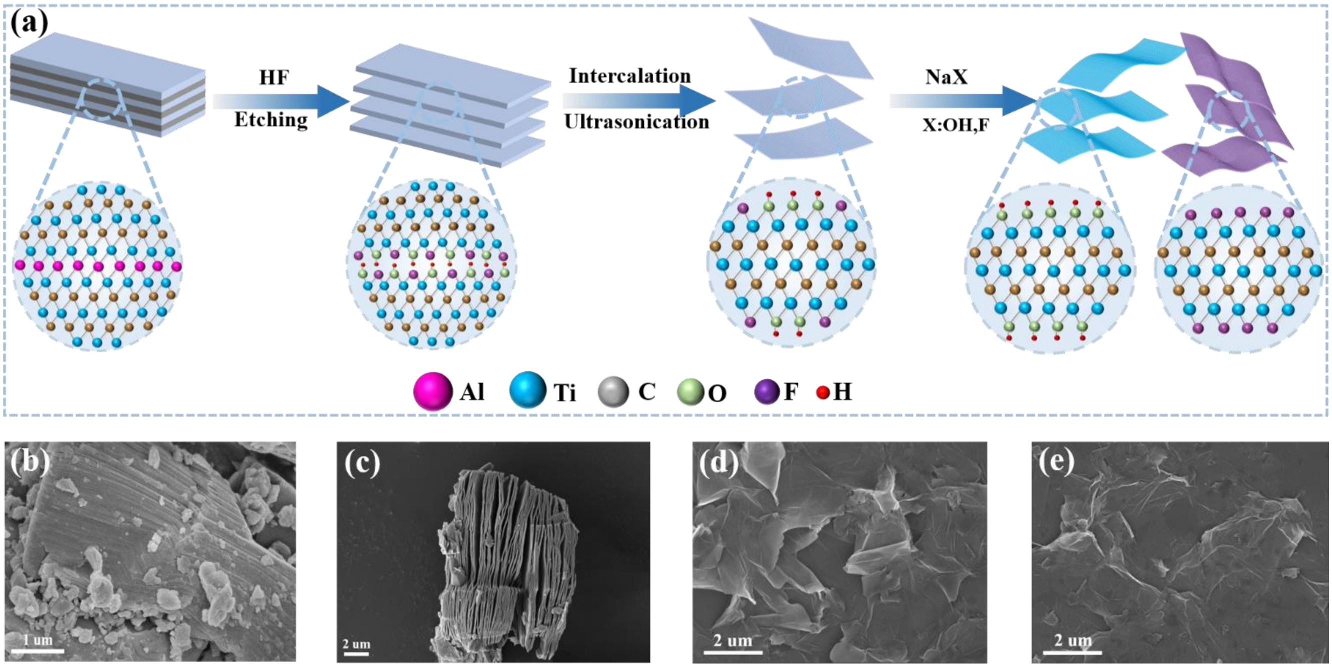

Figure S1. a) Schematic synthetic process of L-Ti_3C_2-R. SEM images of b) Ti_3AlC_2, c) ML-$Ti_3C_2T_x$, d) L-$Ti_3C_2T_x$, and e) L-Ti_3C_2-OH.

MXene nanosheets were prepared by the multi-step process (Figure S1a). In brief, the layered MXene (ML-$Ti_3C_2T_x$, T_x = F or OH) nanostructures were first prepared by HF etching of Ti_3AlC_2 to remove the Al layer. Then the MXene nanosheets (L-$Ti_3C_2T_x$) were obtained by ultrasonic exfoliation, and finally, the surface functionalized MXene nanosheets (L-Ti_3C_2-R, R = OH or F) were obtained by alkalization or fluorination.

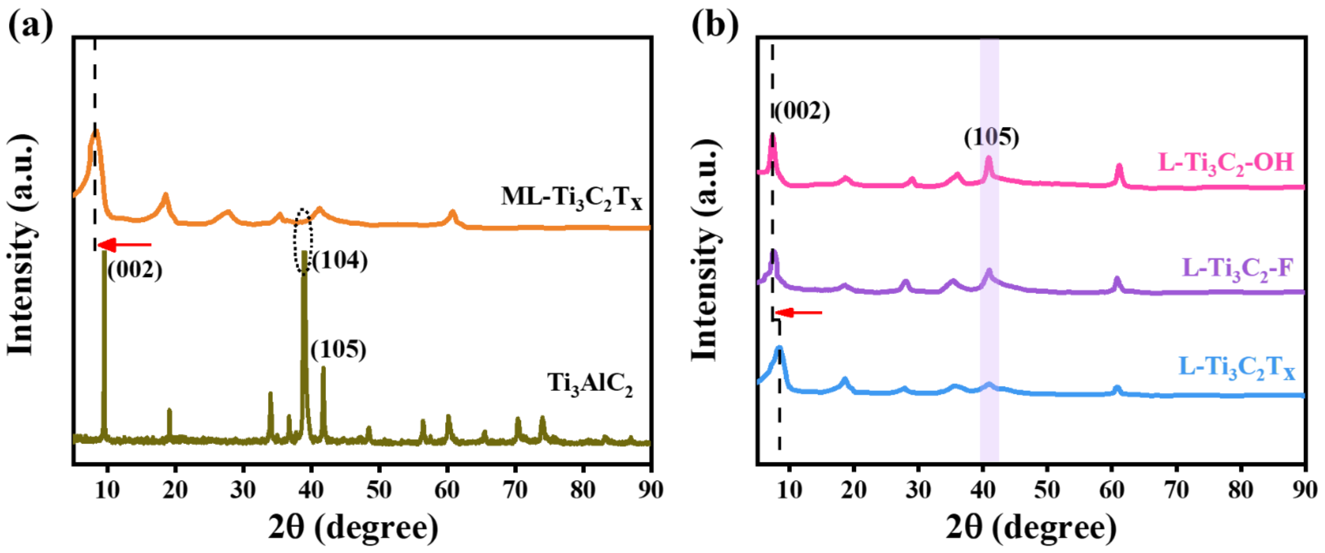

Figure S2. a) XRD patterns of Ti_3AlC_2 and ML-$Ti_3C_2T_x$. b) XRD patterns of L-$Ti_3C_2T_x$, L-Ti_3C_2-F, and L-Ti_3C_2-OH.

In XRD patterns, no diffraction peak corresponding to the (104) crystal plane of the Al layer in ML-$Ti_3C_2T_x$ indicates that Al has been removed after etching.[6] After surface functionalization, the (002) peaks corresponding to the multilayer structure are shifted to a lower angle, which was caused by the increase of the (002) crystal plane spacing of L-$Ti_3C_2T_x$.

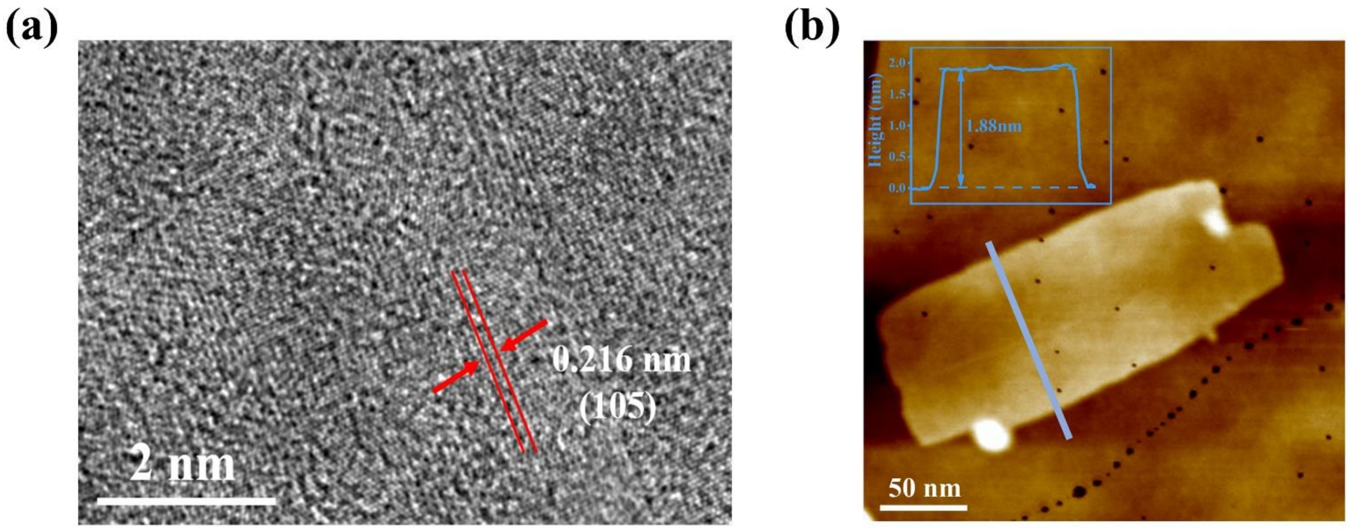

Figure S3. a) HR-TEM image of L-Ti_3C_2-OH. b) AFM image of L-Ti_3C_2-OH.

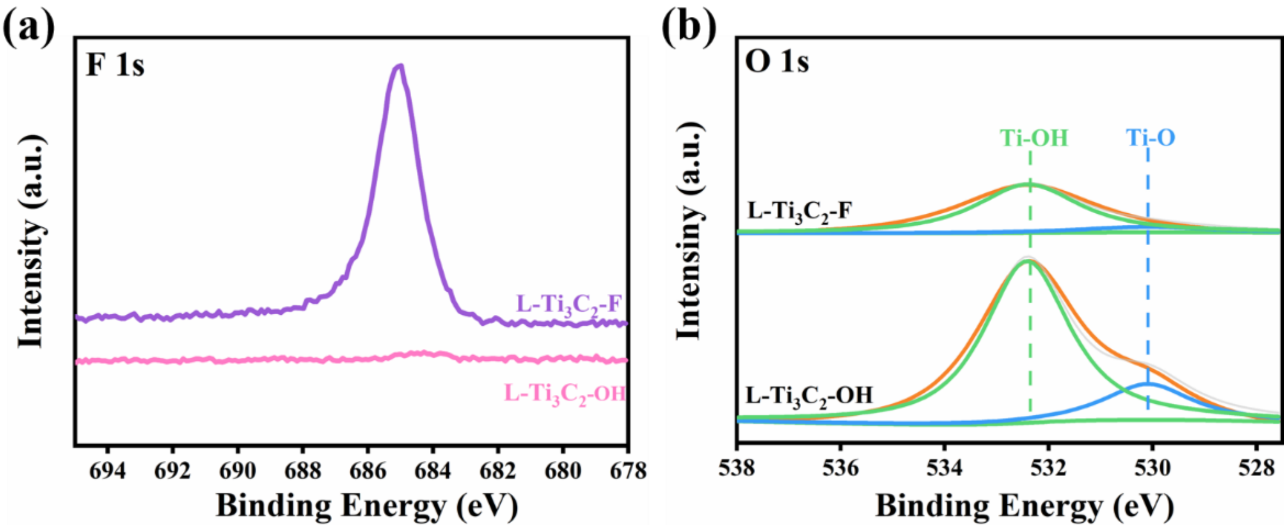

Figure S4. High-resolution core-level XPS spectra of a) F 1s and b) O 1s of L-Ti_3C_2-R.

In XPS spectra, no obvious F-terminating groups were detected for L-Ti_3C_2-OH and the signals of Ti-OH and Ti-O for L-Ti_3C_2-OH are much higher than these of L-Ti_3C_2-F, indicating successful replacement of surface-terminating groups.[7]

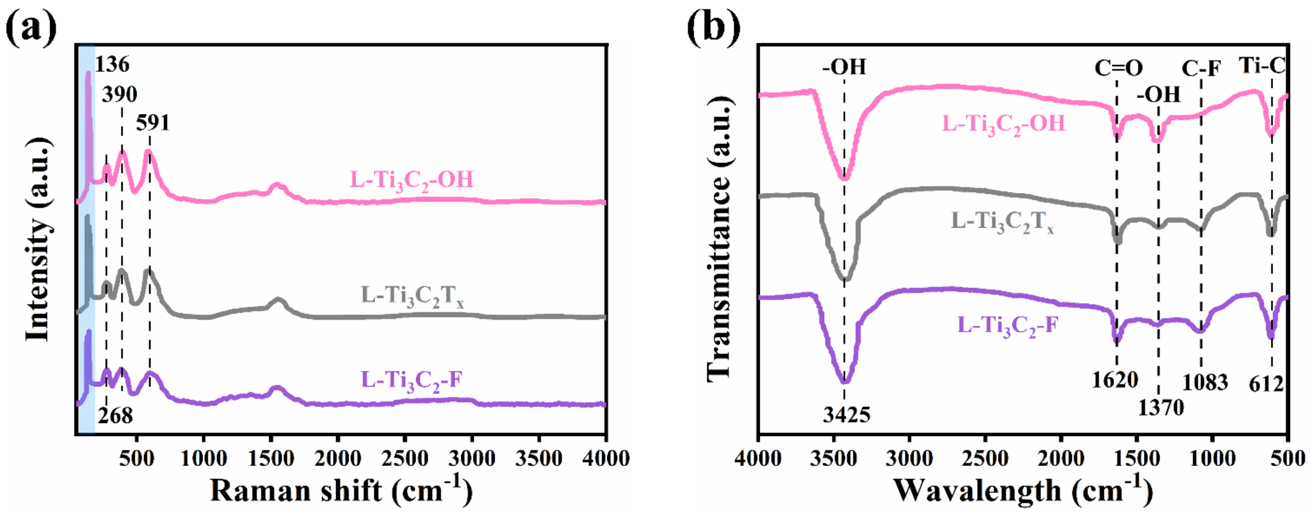

Figure S5. a) Raman patterns of L-$Ti_3C_2T_x$ and L-Ti_3C_2-R. b) FT-IR patterns of L-$Ti_3C_2T_x$ and L-Ti_3C_2-R.

In the Raman spectra, characteristic Raman bands were observed for L-$Ti_3C_2T_x$ (136, 268, 390, and 591 cm^{-1}). For L-Ti_3C_2-OH, the peak intensity attributed to the Ti-O bond (136 cm^{-1}) is significantly higher than that of L-$Ti_3C_2T_x$, illustrating the surface-terminating groups were successfully replaced by -OH, while the peak intensity of L-Ti_3C_2-F is lower than that of L-$Ti_3C_2T_x$, further indicating the abundant

surface-terminating groups were successfully replaced by -F.[8] In Fourier transform infrared (FT-IR) spectroscopy, L-$Ti_3C_2T_x$ and L-Ti_3C_2-R have identical stretching vibrations at about 3425 cm^{-1}, which are attributed to the -OH bond of H_2O.

The lower HOMO and LUMO energy levels of the C-F bond indicate that valence electrons are strongly bound to the nucleus, and the strong electronegativity of fluorine causes it attractive for the lone pair. Therefore, it is hard for F with C-F bonding to provide the lone electron pair to form the axial coordination with the empty d orbital of the metal atom.[9]

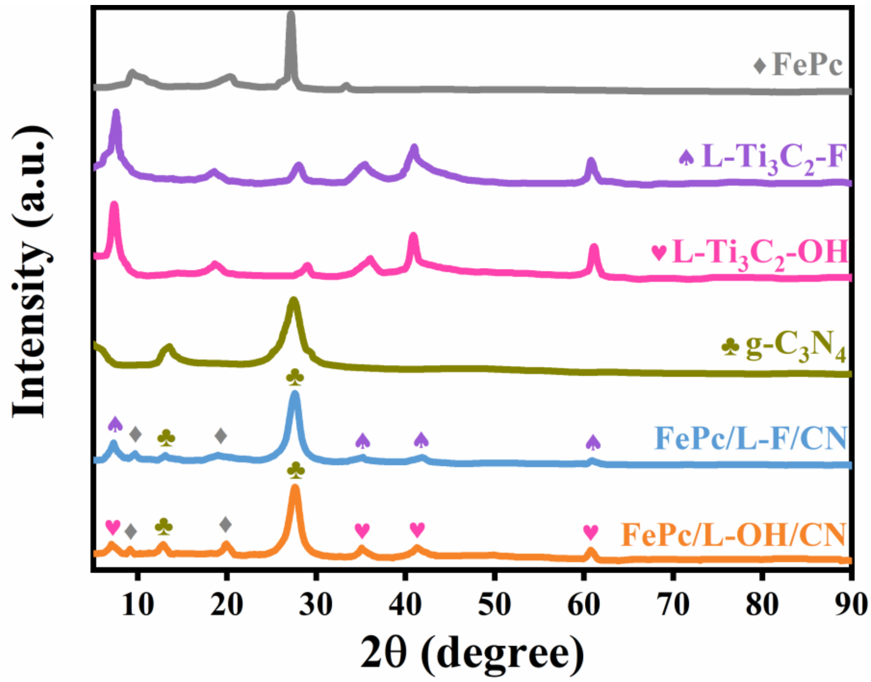

Figure S6. XRD image of FePc, L-Ti_3C_2-R, g-C_3N_4, and FePc/L-R/CN.

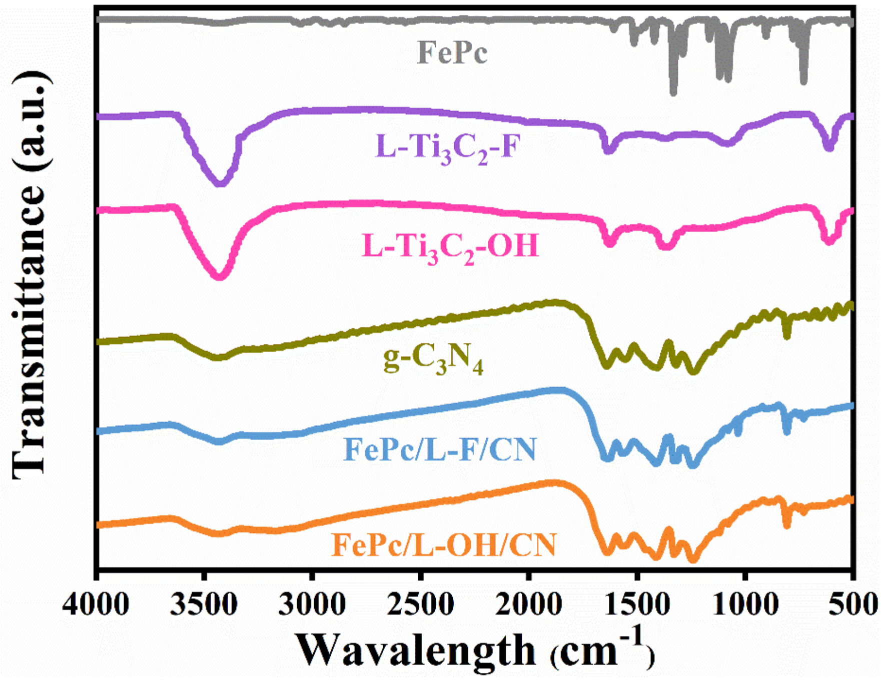

Figure S7. FT-IR patterns of FePc, L-Ti_3C_2-R, g-C_3N_4, and FePc/L-R/CN.

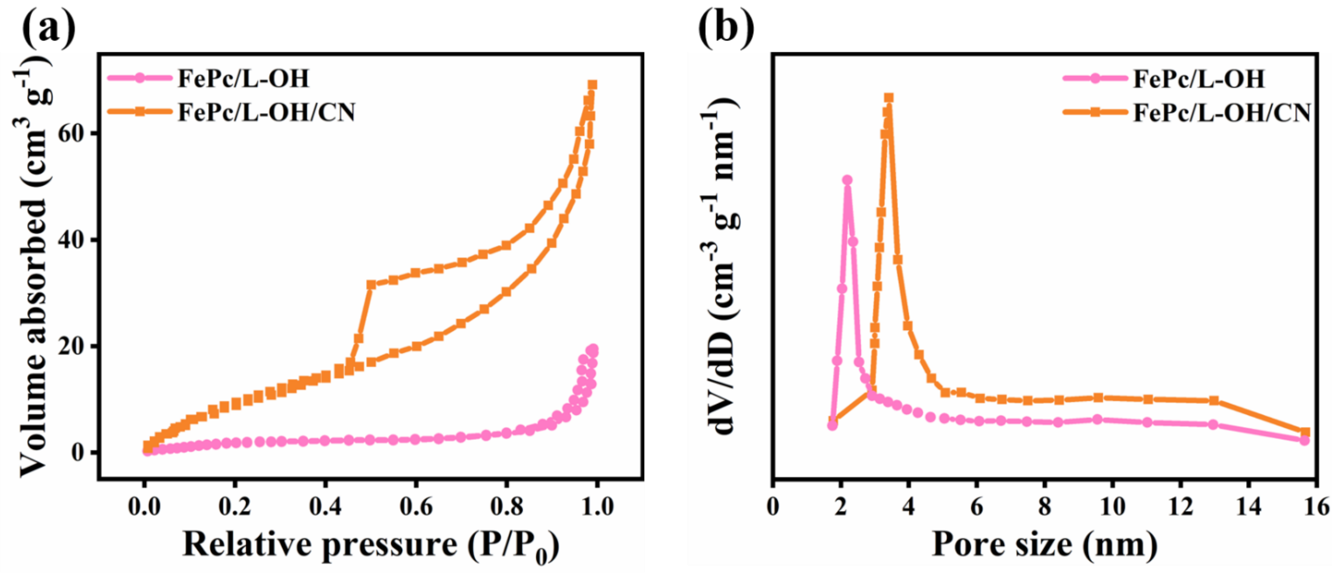

Figure S8. a) N_2 adsorption-desorption isotherms and b) pore-size distributions of FePc/L-OH and FePc/L-OH/CN.

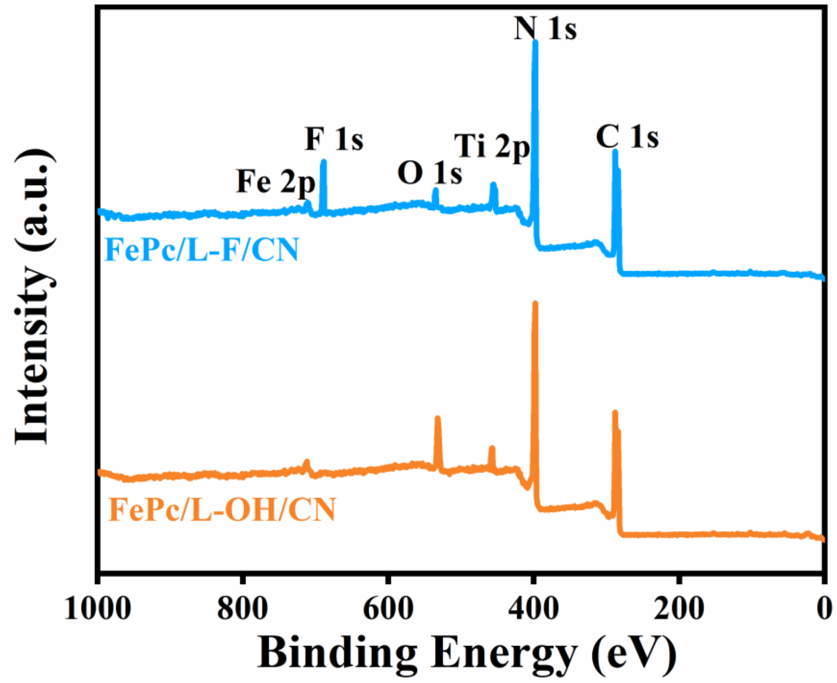

Figure S9. XPS survey spectra of FePc/L-R/CN.

The surface features of the synthesized catalysts were investigated by X-ray photoelectron spectroscopy (XPS) technique. The total spectra of FePc/L-F/CN showed the coexistence of Fe, Ti, C, N, O, and F. In contrast, no signal of F was observed in FePc/L-OH/CN.

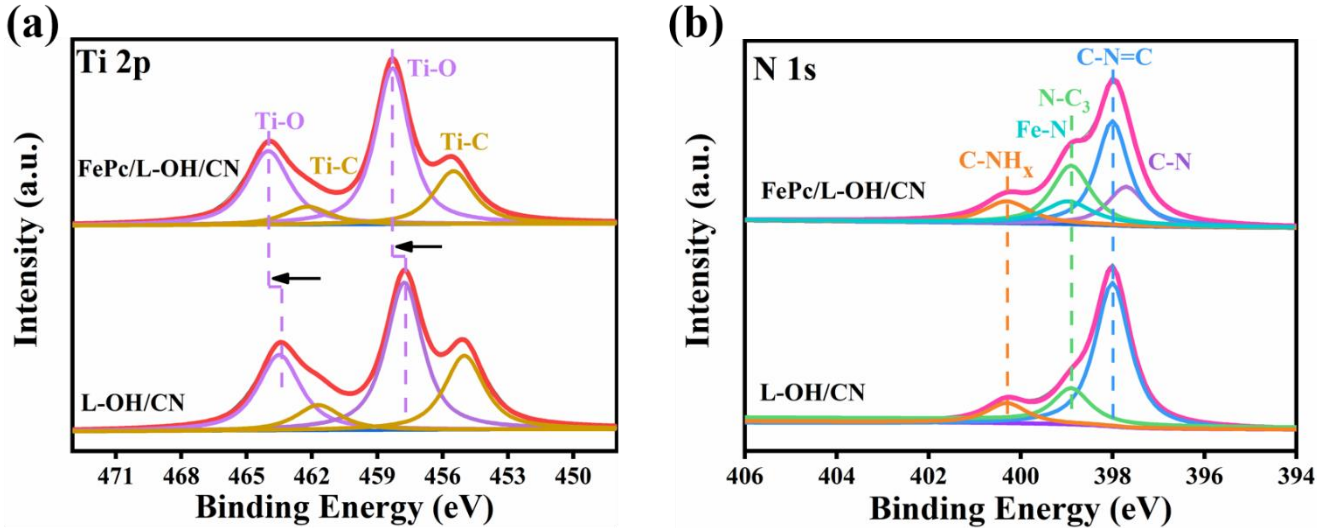

Figure S10. XPS spectra of a) Ti 2p and b) N 1s of L-OH/CN and FePc/L-OH/CN.

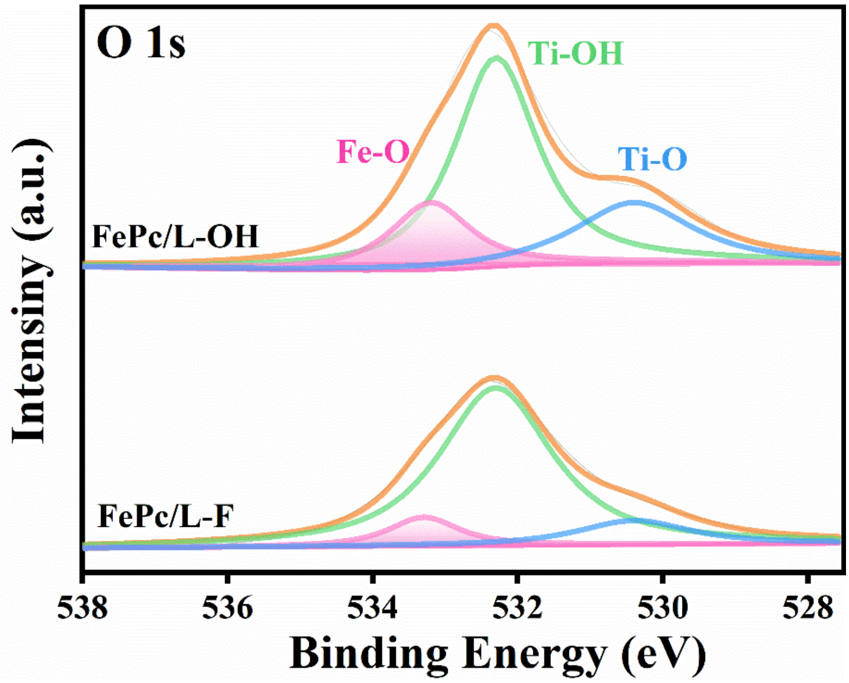

Figure S11. XPS spectra of O 1s for FePc/L-OH and FePc/L-F.

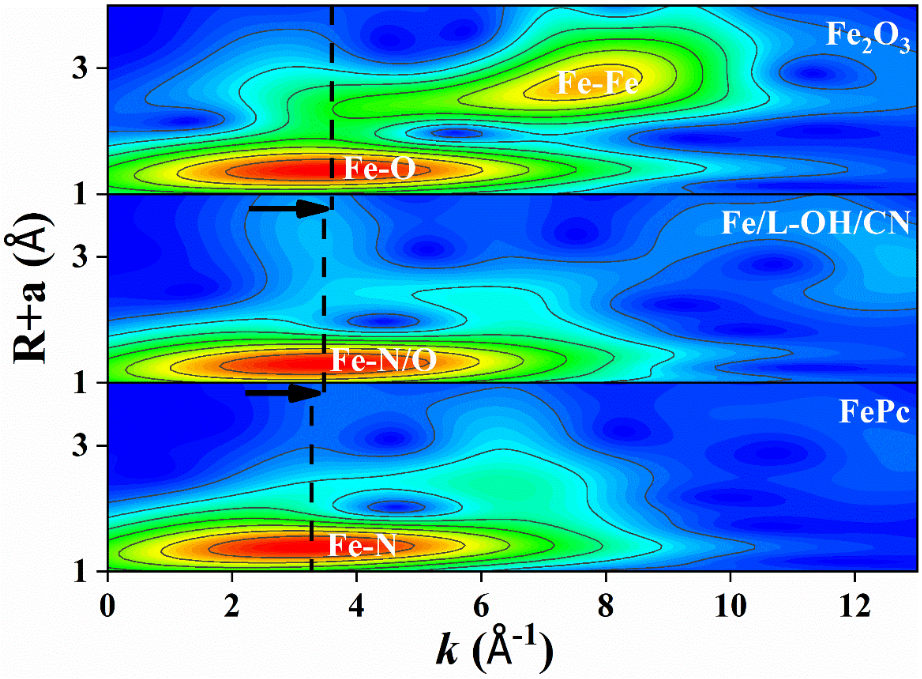

Figure S12. WT-EXANES of Fe_2O_3, FePc, and FePc/L-OH/CN.

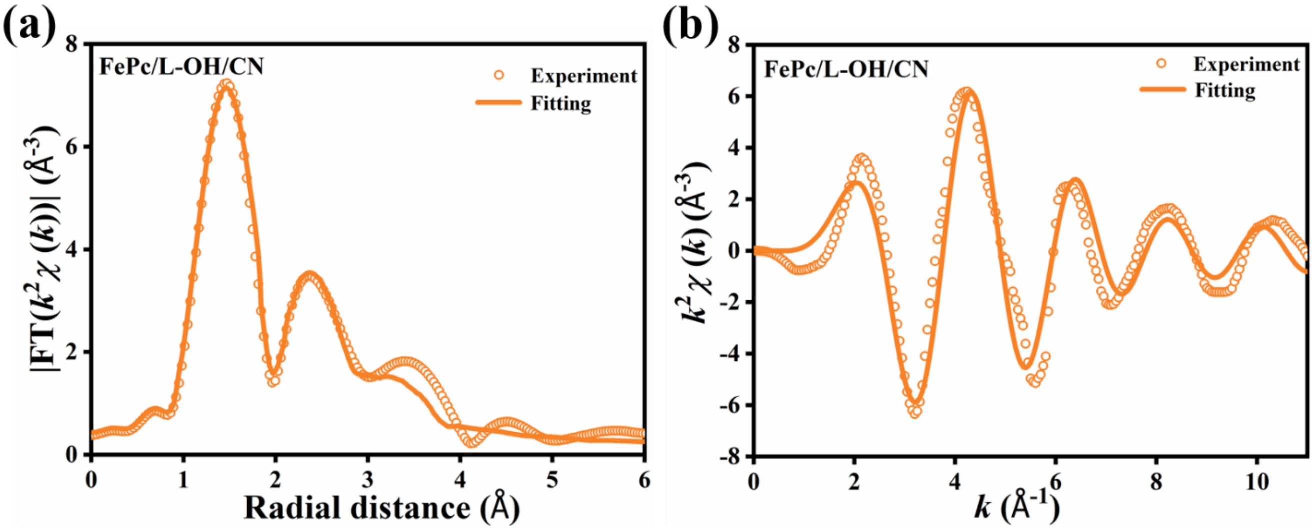

Figure S13. The EXAFS data fitting results of FePc/L-OH/CN.

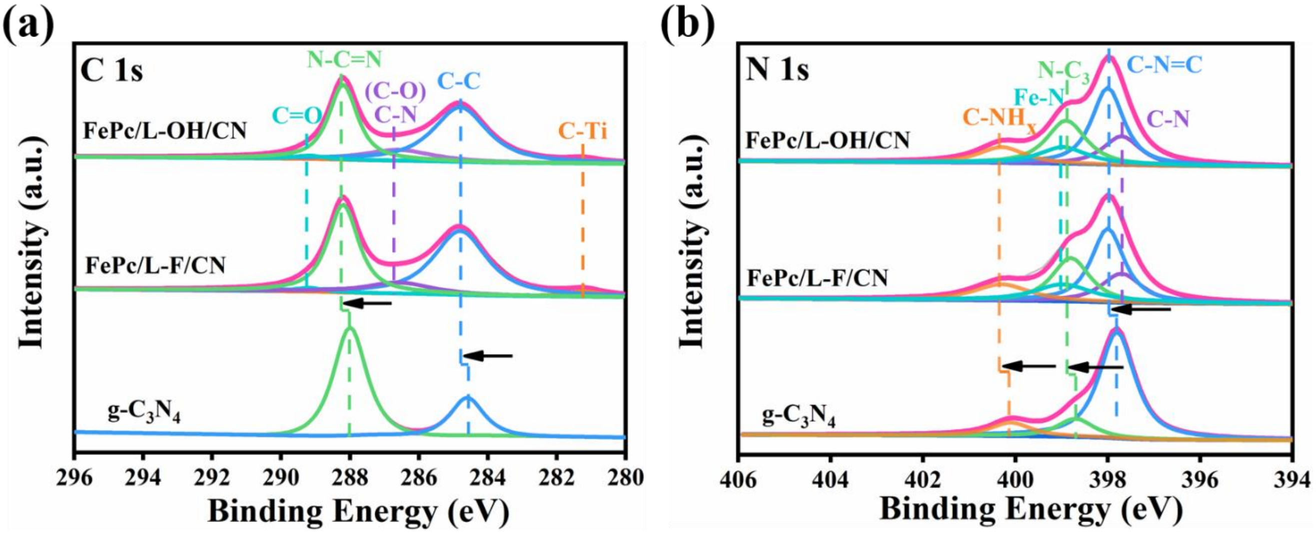

Figure S14. XPS spectra of a) C 1s and b) N 1s of g-C_3N_4 and FePc/L-R/CN.

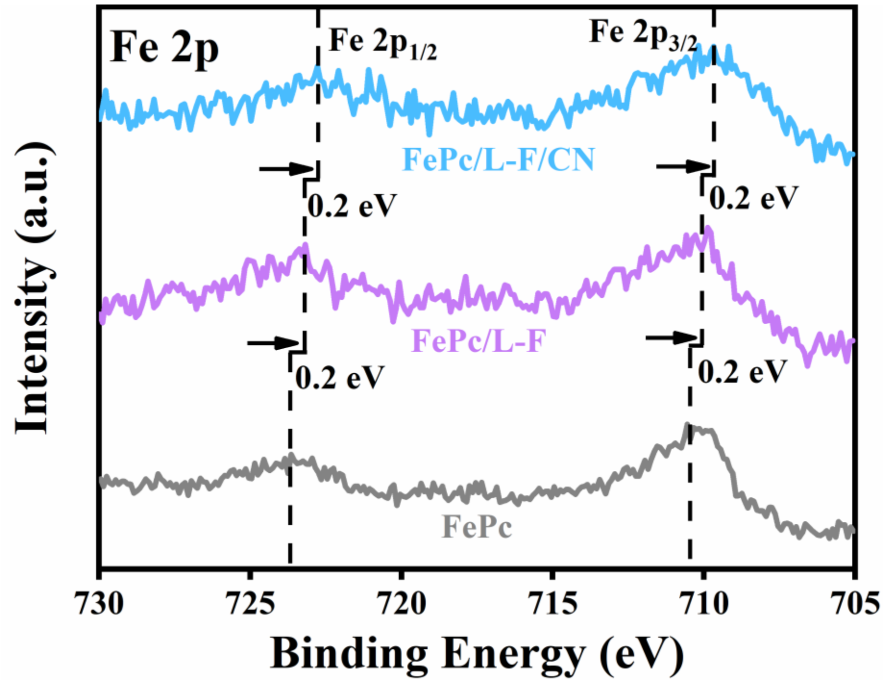

Figure S15. XPS spectra of Fe 2p for FePc, FePc/L-F, and FePc/L-F/CN.

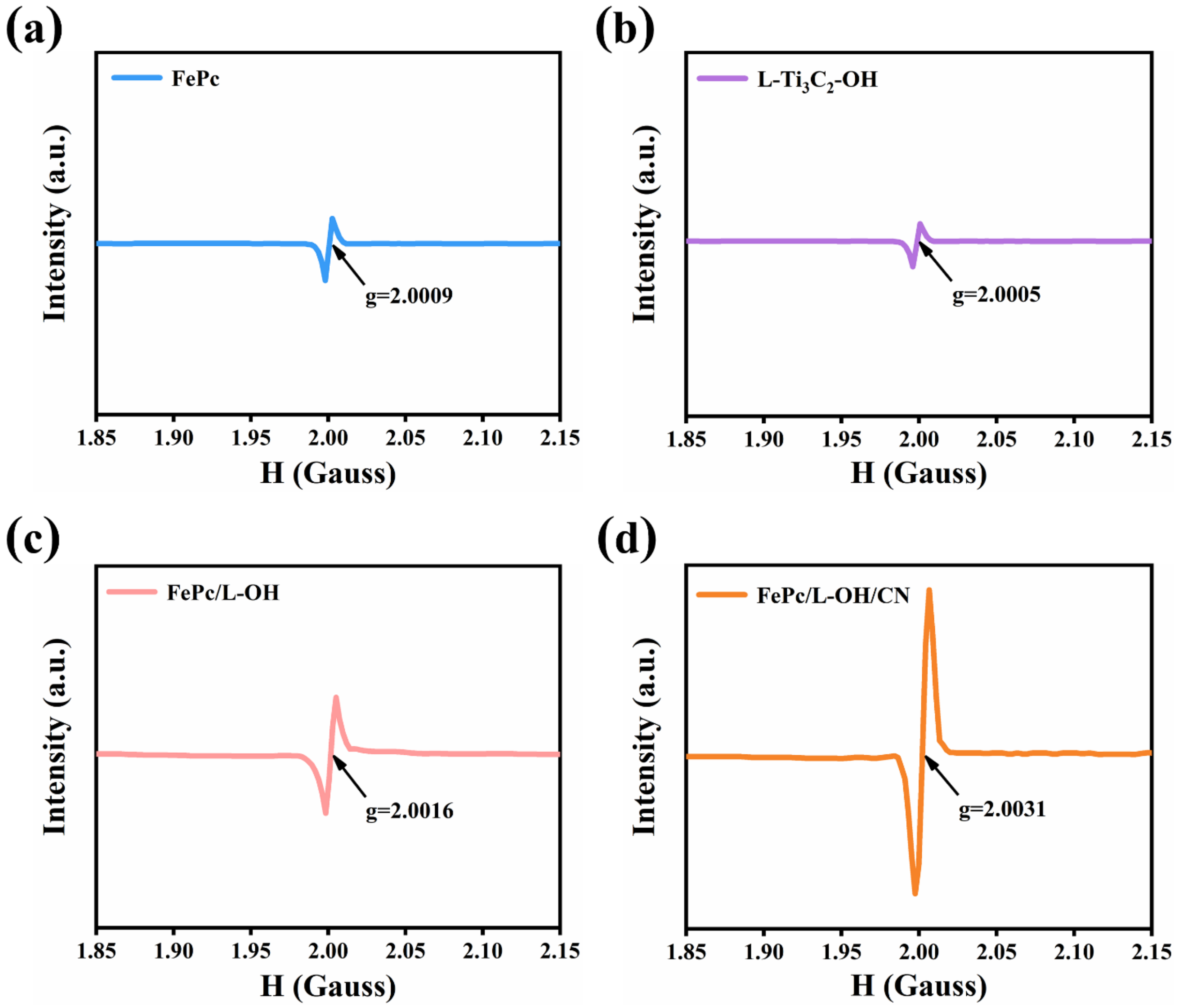

Figure S16. The EPR spectra with g-factor values of a) FePc, b) L-Ti_3C_2-OH, c) FePc/L-OH, and d) FePc/L-OH/CN.

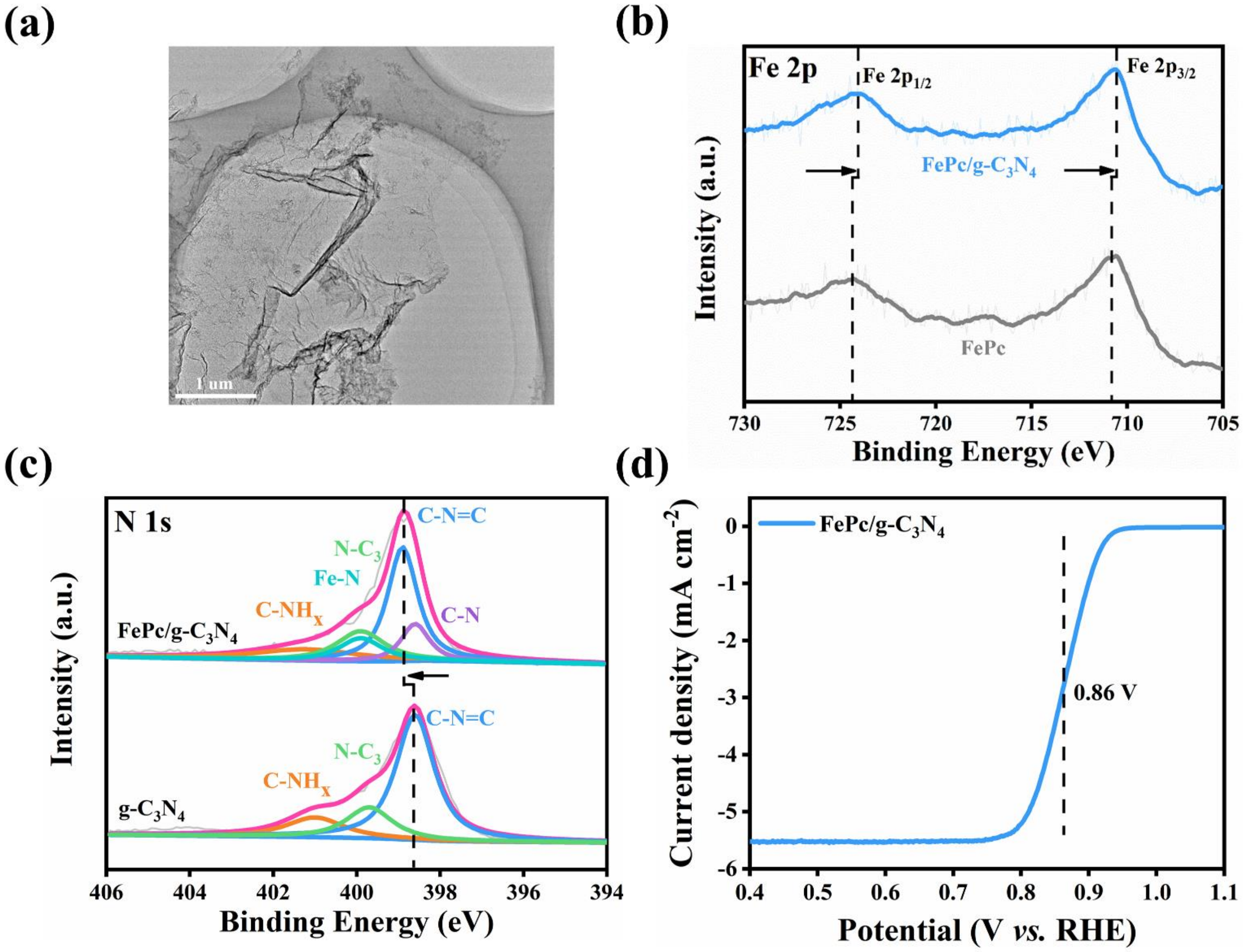

Figure S17. a) TEM image of FePc/g-C_3N_4. b) XPS spectra of Fe 2p for FePc and FePc/g-C_3N_4. c) XPS spectra of N 1s for g-C_3N_4 and FePc/g-C_3N_4. d) LSV curve of FePc/g-C_3N_4.

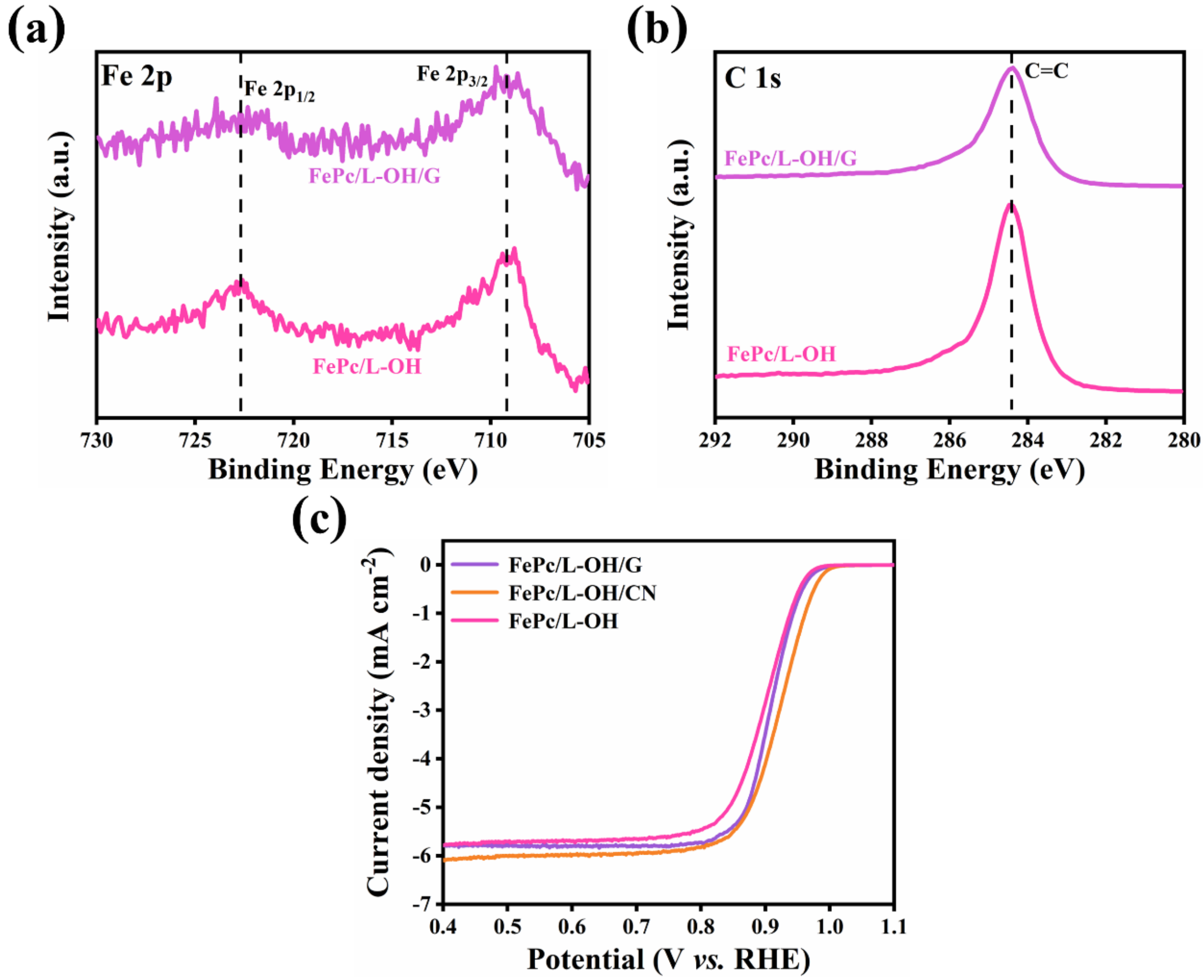

Figure S18. XPS spectra of a) Fe 2p and b) C 1s for FePc/L-OH and FePc/L-OH/G. c) LSV curves of FePc/L-OH, FePc/L-OH/CN, and FePc/L-OH/G.

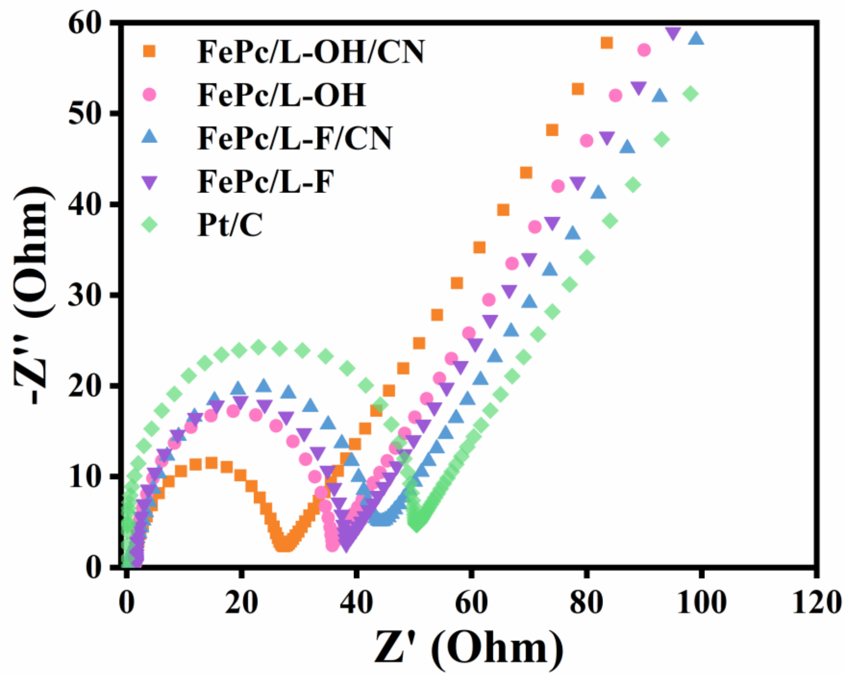

Figure S19. EIS impedance test chart of FePc/L-R, FePc/L-R/CN, and Pt/C.

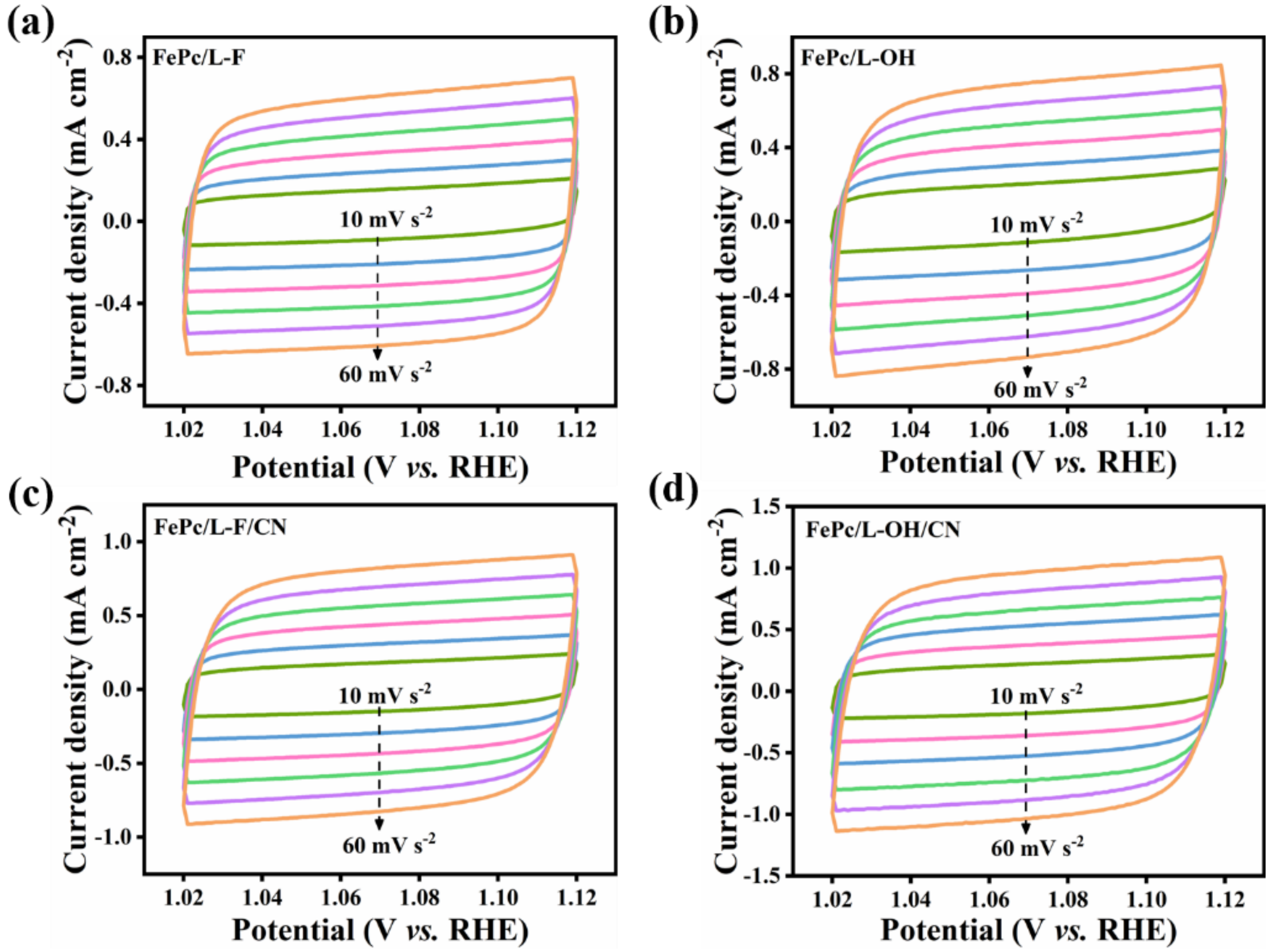

Figure S20. Cyclic voltammograms measurements of a) FePc/L-F, b) FePc/L-OH, c) FePc/L-F/CN, and

d) FePc/L-OH/CN at various scan rates (10, 20, 30, 40, 50, and 60 mV s^{-1}).

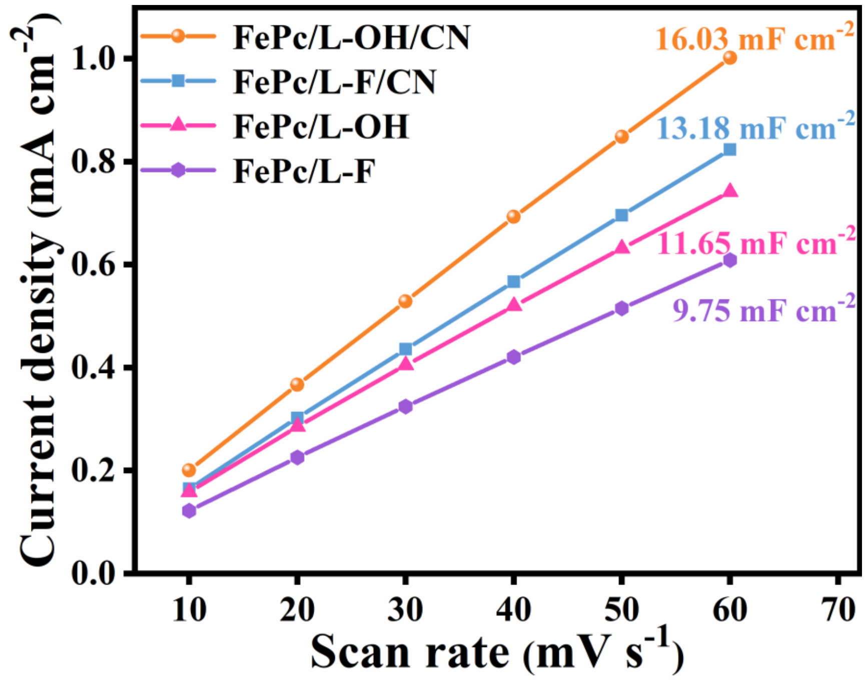

Figure S21. Relationship between scan rate and current density at 1.05 V derived from CV curves, and corresponding C_{dl} value of catalysts.

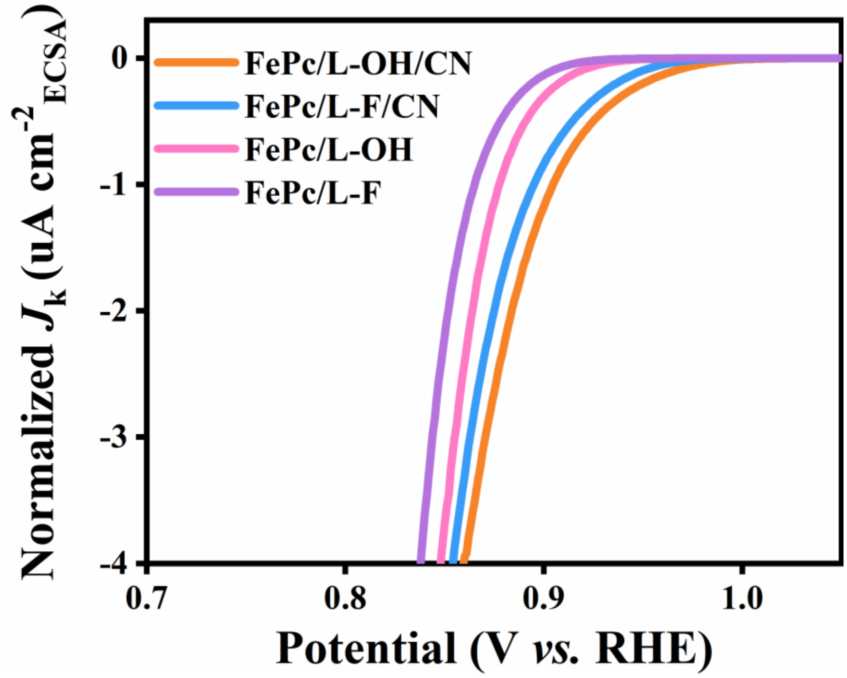

Figure S22. Normalized kinetic current densities by ECSA of FePc/L-OH/CN, FePc/L-F/CN,

FePc/L-OH, and FePc/L-F.

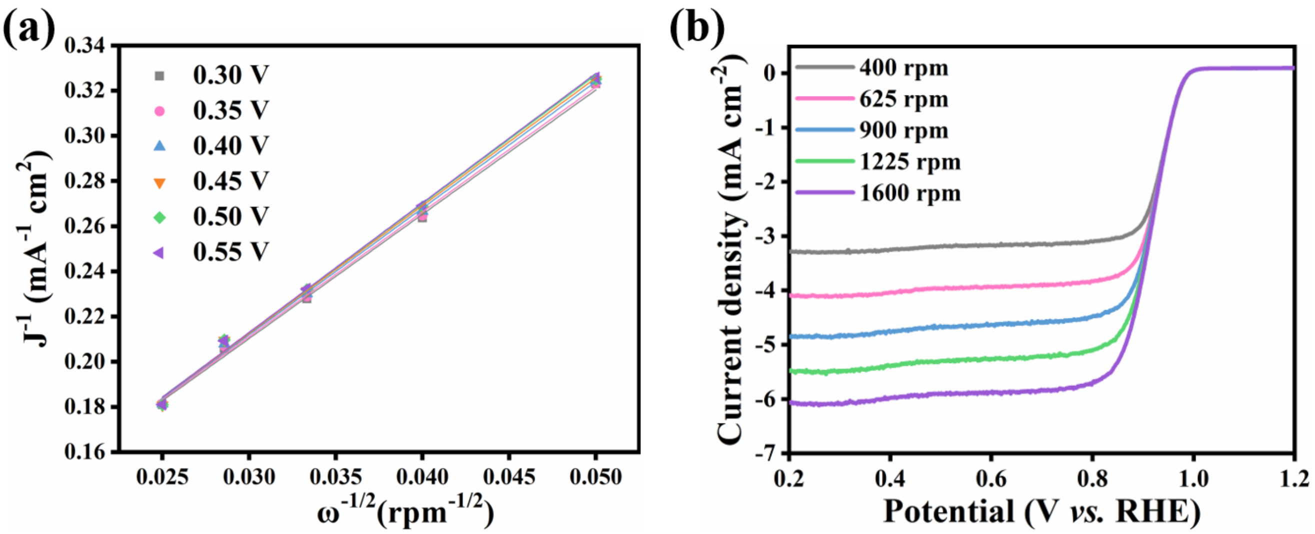

Figure S23. LSV curves and K-L curves of FePc/L-OH/CN at different speeds in 0.1 M KOH solution.

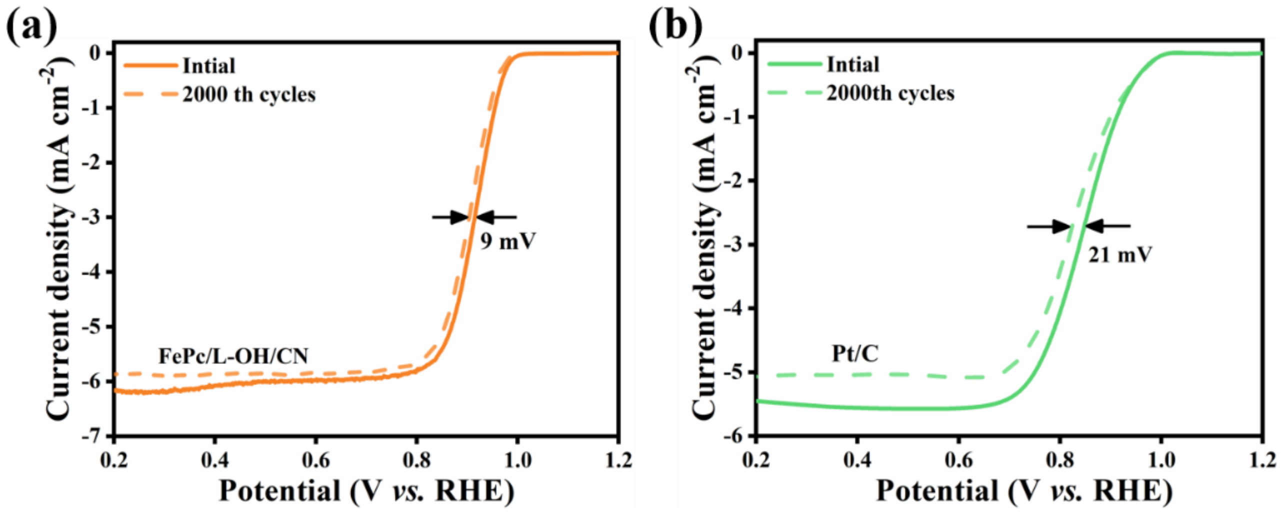

Figure S24. Stability test: LSV curves of a) FePc/L-OH/CN and b) Pt/C before/after 2 000 CV cycles.

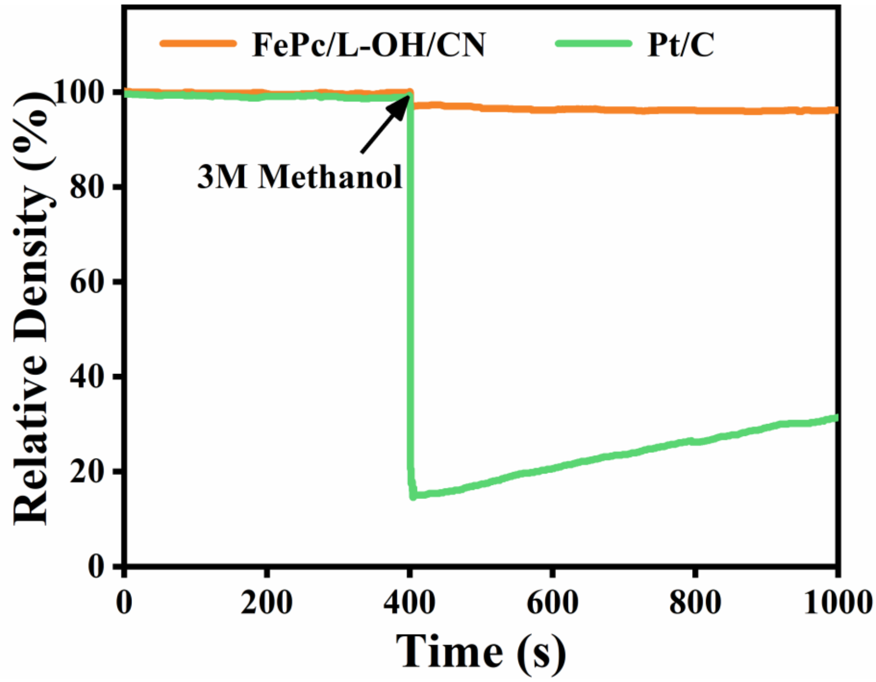

Figure S25. I-t curves of Pt/C and FePc/L-OH/CN for the methanol-tolerance test.

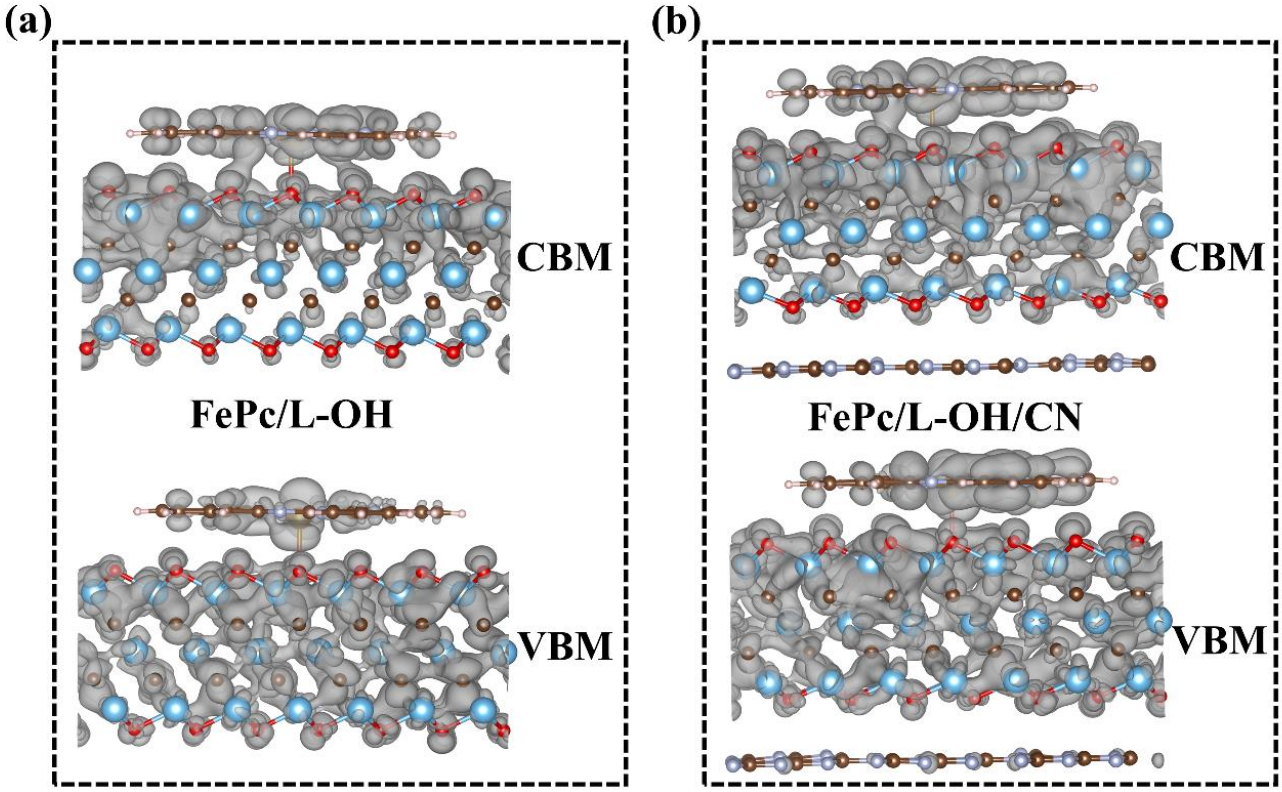

Figure S26. The DFT calculations of the electronic structure of a) FePc/L-OH and b) FePc/L-OH/CN with corresponding charge distribution of VBM and CBM.

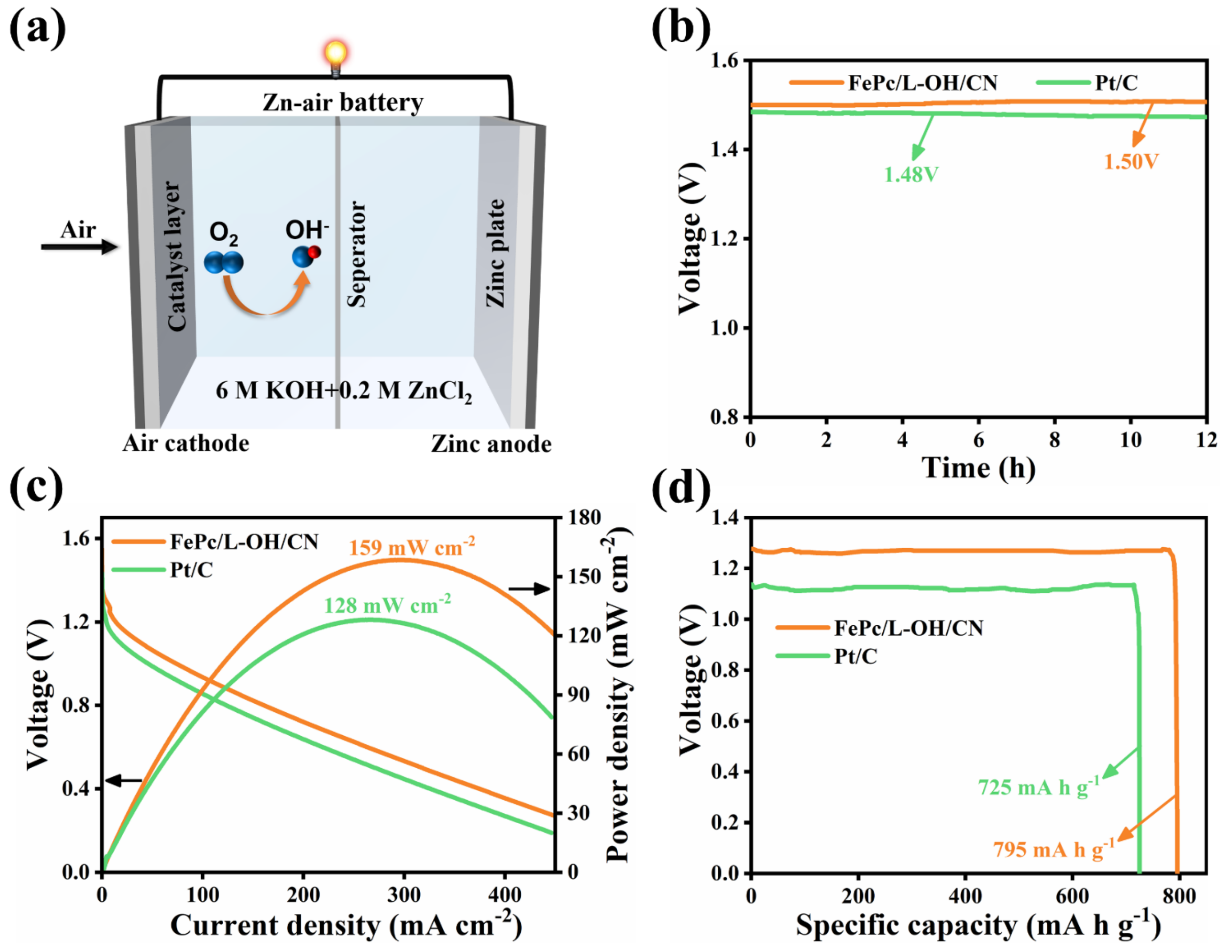

Figure S27. a) Schematic illustration of a typical ZAB. b) Open circuit potential. c) Polarization curves and the corresponding power density curves. d) Specific capacity.

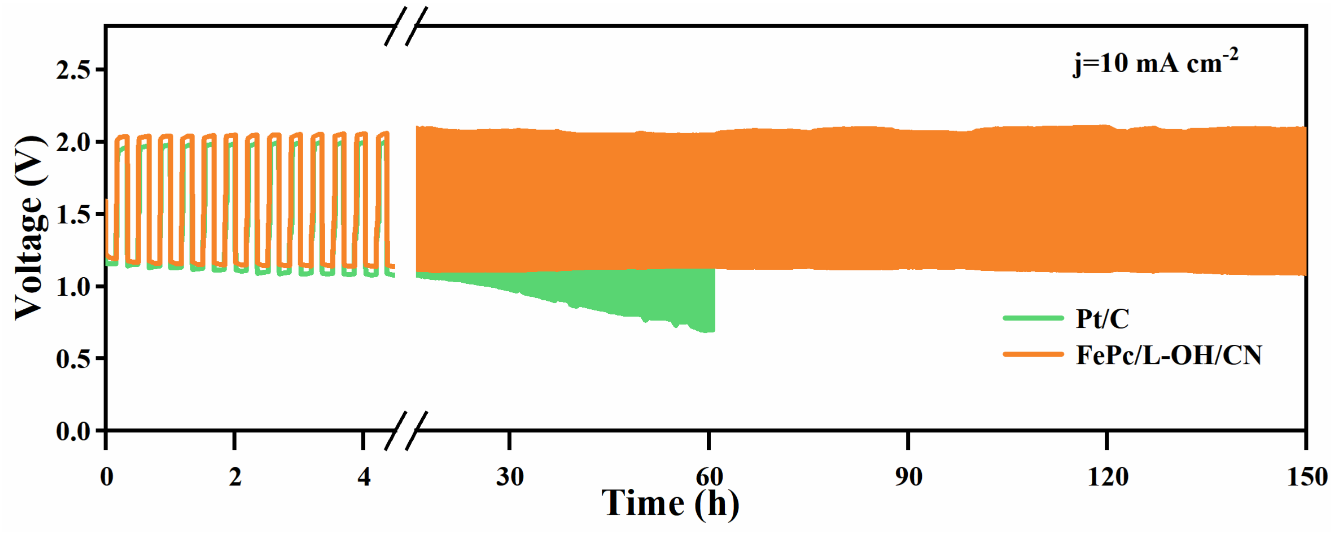

Figure S28. Long-term galvanostatic charge-discharge test of FePc/L-OH/CN-based ZAB at a current

density of 10 mA cm^{-2}.

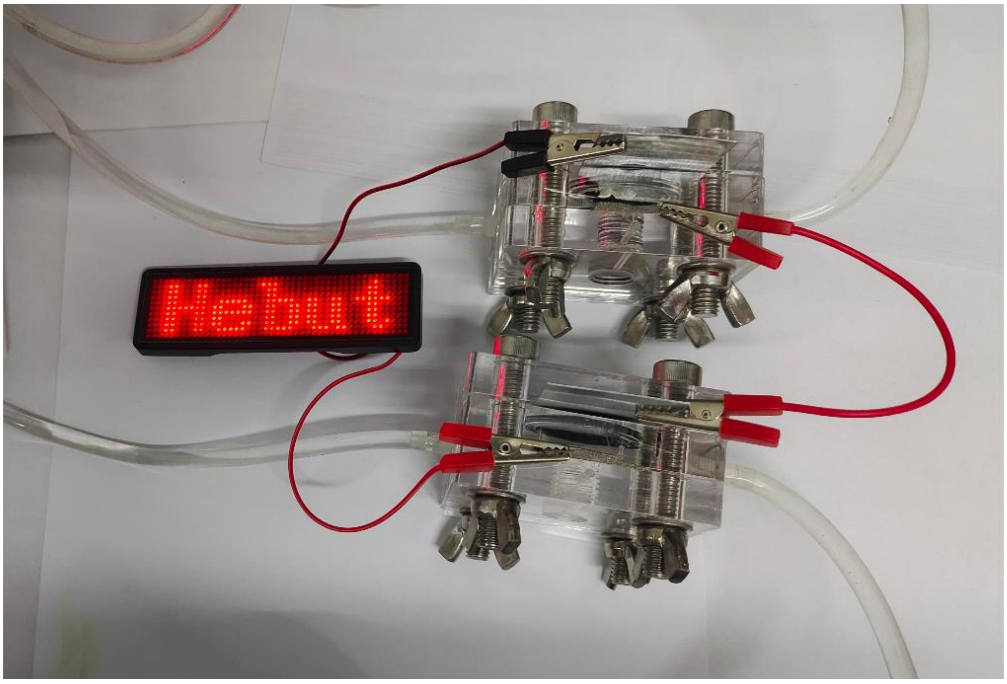

Figure S29. The LED is powered by the FePc/L-OH/CN-based ZAB.

Table S1. EXAFS fitting parameters at the Fe K-edge for various samples（S_0^2=0.884）

Sample	Shell	CN^a	R(Å)^b	σ^2(Å^2)^c	ΔE_0(eV)^d	R factor
Fe foil	Fe-Fe	8*	2.451	0.0036	3.7	0.0029
	Fe-Fe	6*	2.841	0.0027		
FePc	Fe-N	4.00	1.951	0.0099	4.1	0.0020
Fe_2O_3	Fe-O	6.00	1.953	0.0125	−5.7	0.0075
FePc/L-OH/CN	Fe-N	3.80	1.920	0.0021	0.5	0.0033
	Fe-O	1.10	2.134			

^{a}*CN*, coordination number; ^{b}*R*, the distance to the neighboring atom; $^c\sigma^2$, the Mean Square Relative Displacement (MSRD); $^d\Delta E_0$, inner potential correction; *R* factor indicates the goodness of the fit. S_0^2 was fixed to 0.884, according to the experimental EXAFS fit of Fe foil by fixing *CN* as the known crystallographic value. * This value was fixed during EXAFS fitting, based on the known structure of Fe. Fitting range: $3.0 \leq k$ (Å) ≤ 11.7 and $1.0 \leq R$ (Å) ≤ 3.0 (Fe foil); $1.0 \leq k$ (Å) ≤ 9.5 and $1.0 \leq R$ (Å) ≤ 3.0 (Sample Fe). A reasonable range of EXAFS fitting parameters: $0.700 < S_0^2 < 1.000$; $CN > 0$; $\sigma^2 > 0$ Å^2; $|\Delta E_0| < 10$ eV; R factor < 0.02.

Table S2. Comparison of ORR performance between FePc/L-OH/CN and state-of-the-art Fe-N-C

catalysts reported in the literature.

Electrocatalysts	E_{onset} (V)	$E_{1/2}$ (V)	Reference
FePc/L-OH/CN	1.02	0.92	**This work**
FePc/$Ti_3C_2T_x$	0.97	0.89	*Adv. Mater*. **2018**, *30,* 1803220.
FePc/1L-Ti_3C_2	1.0	0.92	*J. Mater. Chem. A.* **2022**, *10*, 24041.
Alk-MXene/FePc	1.0	0.924	*Adv. Mater*. **2023**, *15,* 2210757.
Fe_{MC}-MXene/GrH	1.02	0.96	*Adv. Funct. Mater.* **2022**, *33,* 2210101.
FeAB-O	0.98	0.90	*Nat. Commun.* **2020**, *11,* 4173.
FePc/DG	0.98	0.90	*Appl. Catal. B* **2021**, *280*, 119437.
FePc/CNT-NH_2	1.0	0.92	*ChemSusChem.* **2021**, *15,* e202102379.
o-MQFe-10: 20: 5	0.96	0.86	*Angew. Chem. Int. Ed.* **2022**, *61*, e202117617.
SNGO/FePc	1.02	0.94	*ChemSusChem*. **2022**, *15,* e202200195.
Fe-N/P-C-700	0.94	0.87	*J. Am. Chem. Soc.* **2020**, *142,* 2404.
FePc&rGO	0.98	0.89	*Energy Storage Mater*. **2022**, *50,* 12.
FeN_4-$Ti_3C_2S_x$	1.01	0.89	*Nano-Micro Lett.* **2023**, *15,* 47.
FePc-Py-CNTs	0.98	0.91	*Nat. Commun.* **2013**, *4*, 2076.
Fe-N-C@$Ti_3C_2T_x$	-----	0.88	*J. Mater. Chem. A.* **2021**, *9*, 3952.

References

[1] J. Hutter, M. Iannuzzi, F. Schiffmann, J. VandeVondele, *Mol. Sci.* **2014**, *4*, 15.

[2] B.G. LIPPERT, J. HUTTER, M. PARRINELLO, *Mol. Phys.* **1997**, *92*, 477.

[3] J. VandeVondele, J. Hutter, *J. Chem. Phys.* **2007**, *127*, 114105.

[4] S. Goedecker, M. Teter, J. Hutter, *Phys. Rev. B.* **1996**, *54*, 1703.

[5] S. Grimme, J. Antony, S. Ehrlich, H. Krieg, *J. Chem. Phys.* **2010**, *132*, 154104.

[6] Z. Li, Z. Zhuang, F. Lv, H. Zhu, L. Zhou, M. Luo, J. Zhu, Z. Lang, S. Feng, W. Chen, L. Mai, S. Guo, *Adv. Mater.* **2018**, *30*, 1803220.

[7] Y. Dai, B. Liu, Z. Zhang, P. Guo, C. Liu, Y. Zhang, L. Zhao, Z. Wang, *Adv. Mater.* **2023**, *35*, 2210757.

[8] J. Xia, S.-Z. Yang, B. Wang, P. Wu, I. Popovs, H. Li, S. Irle, S. Dai, H. Zhu, *Nano Energy* **2020**, *72*, 104681.

[9] F. Yu, Z. Zhou, Y. You, J. Zhan, T. Yao, L. H. Zhang, *ACS Appl. Mater. Interfaces* **2023**, *15*, 24346.
